# Cancer-associated Notch receptor variants lead to *O*-fucosylation defects that deregulate Notch signaling

**DOI:** 10.1016/j.jbc.2022.102616

**Published:** 2022-10-18

**Authors:** Florian Pennarubia, Atsuko Ito, Megumi Takeuchi, Robert S. Haltiwanger

**Affiliations:** Complex Carbohydrate Research Center, Department of Biochemistry and Molecular Biology, University of Georgia, Athens, Georgia, USA

**Keywords:** Notch, EGF repeat, *O*-fucosylation, cancer, POFUT1, CHO, Chinese hamster ovary, DLL, Delta-like ligand, EGF, epidermal growth factor–like repeat, EV, empty vector, GOF, gain of function, JAG, JAGGED, LFNG, LUNATIC FRINGE, LOF, loss of function, MS, mass spectrometry, N1, NOTCH1, NECD, Notch extracellular domain, PDB, Protein Data Bank, PE, phycoerythrin, POFUT1, Protein *O*-fucosyltransferase 1, RFP, red fluorescent protein

## Abstract

NOTCH1 is a transmembrane receptor that initiates a signaling pathway involved in embryonic development of adult tissue homeostasis. The extracellular domain of NOTCH1 is composed largely of epidermal growth factor–like repeats (EGFs), many of which can be *O*-fucosylated at a specific consensus sequence by protein *O*-fucosyltransferase 1 (POFUT1). *O*-fucosylation of NOTCH1 is necessary for its function. The Notch pathway is deregulated in many cancers, and alteration of POFUT1 has been reported in several cancers, but further investigation is needed to assess whether there is deregulation of the Notch pathway associated with mutations that affect *O*-fucosylation in cancers. Using Biomuta and COSMIC databases, we selected nine NOTCH1 variants that could cause a change in *O*-fucosylation of key EGFs. Mass spectral glycoproteomic site mapping was used to identify alterations in *O*-fucosylation of EGFs containing the mutations. Cell-based NOTCH-1 signaling assays, ligand-binding assays, and cellsurface analysis were used to determine the effect of each mutation on Notch activation. Two variants led to a gain of function (GOF), six to a loss of function (LOF), and one had minimal effects. Most GOF and LOF were associated with a change in *O*-fucosylation. Finally, by comparing our results with known NOTCH1 alterations in cancers from which our mutations originated, we were able to establish a correlation between our results and the known GOF or LOF of NOTCH1 in these cancers. This study shows that point mutations in N1 can lead to alterations in *O*-fucosylation that deregulate the Notch pathway and be associated with cancer processes.

The Notch signaling pathway regulates developmental processes and the maintenance of homeostasis in adult tissues (1, 2). NOTCH1 (N1) is a transmembrane receptor and one of four Notch receptors found in mammals (N1–4) (3). The ability of N1 to induce signaling depends on its interaction with canonical ligands, Delta-like ligands (DLL1 and 4) or JAGGED (JAG1 and 2). This interaction induces a conformational change in the receptor because of a pulling force exerted by ligand endocytosis, allowing the proteolytic release of the Notch intracellular domain, which subsequently translocates to the nucleus to induce transcription of Notch target genes (3). Deregulation of the Notch pathway is associated with many cancers (4, 5). Interestingly, Notch signaling can be oncogenic or tumor suppressive depending on the context and tissue (4, 5). Thus, a loss of function (LOF) of N1 has been found in various squamous cell carcinomas (6, 7, 8, 9), carcinomas (8), and low-grade glioma (10). Conversely, a gain of function (GOF) of N1 has been found in various lymphomas/leukemia (11, 12, 13) and breast cancer (14). Glycosylation of the Notch extracellular domain (NECD) regulates its activity and is essential for its function, and alterations in the glycosyltransferases modifying the NECD have been implicated in a number of cancers (15, 16).

The NECD contains 36 tandem epidermal growth factor–like (EGF) repeats. Each EGF consists of ∼40 amino acids and characterized by the presence of six conserved cysteines connected by three disulfide bonds (C^1^-C^3^, C^2^-C^4^, and C^5^-C^6^) (17). Properly folded EGFs of N1 are modified by multiple protein *O-*glycosyltransferases (18). Protein *O-*fucosyltransferase 1 (POFUT1) (19, 20) catalyzes the transfer of a fucose to the hydroxyl of a serine or a threonine in the consensus sequence C^2^XXXX(S/T)C^3^ (21, 22). After transfer, the *O-*fucose can be extended by a GlcNAc *via* the Fringe family of enzymes to form a disaccharide. The Fringe family comprises three members, LUNATIC, MANIC, and RADICAL, of which LUNATIC FRINGE (LFNG) is the most effective (23, 24, 25). The disaccharide can be further elongated to trisaccharide or tetrasaccharide by the respective addition of a galactose and a sialic acid (23, 26).

The presence of an *O-*fucose on N1 is essential for its trafficking and function; this is particularly true for EGF8 and EGF12 in the ligand-binding domain (Fig. 1*A*) (23, 27, 28, 29, 30). Cocrystallization of a portion of the N1 ligand–binding domain with its ligands DLL4 or JAG1 showed a direct interaction of *O-*fucose on EGF8 and EGF12 with ligand. The *O-*fucose on EGF12 from N1 directly contacts DLL4 and JAG1, whereas the *O*-fucose on EGF8 directly interacts with JAG1 (31, 32). Loss of *O*-fucose on EGF8 or EGF12 decreases binding of N1 to its ligands resulting in decreased associated signaling (23, 32). Elimination of *O*-fucose on EGF8 also led to a decrease in N1 expression at the cell surface (23). While the *O-*fucose on EGF9 is not directly involved in interaction with ligands, elimination of *O*-fucose on EGF9 also induces a decrease in ligand binding because of a decrease in expression at the cell surface (23, 33).

**Figure 1:**
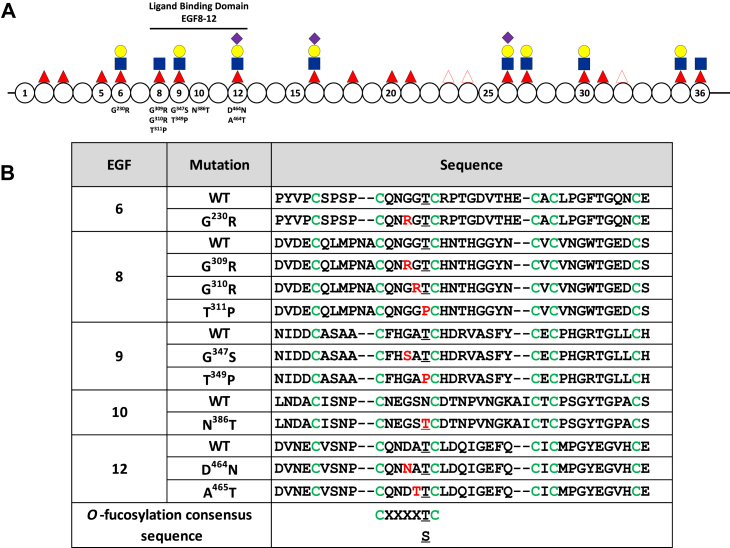
**N1 extracellular domain and position of the mutations selected for analysis.***A*, representation of the extracellular part of the N1 receptor containing EGF1 to 36. EGFs indicated by *circles*, and selected EGFs have numbers inside. The ligand-binding domain comprising EGF8 to 12 is shown. The positions of *O*-fucoses and their elongation by LFNG based on Refs. (23,24). Fucose, *filled red triangles*; GlcNAc, *filled blue square*; galactose, *filled yellow circle*; and sialic acid, *filled purple diamond*. The *empty red triangle* represents unoccupied *O*-fucosylation sites. The position of each mutation is below its respective EGF. *B*, peptide sequence of the WT and mutant for each EGF used in this study. Position of each mutation in their respective EGF is highlighted in *red*. Cysteines are colored *green*, and *O*-fucosylation sites are *underlined*. *O*-fucosylation consensus sequence is shown at the *bottom*. EGF, epidermal growth factor–like repeat; LFNG, LUNATIC FRINGE; N1, NOTCH1.

Extension of *O-*fucose by FRINGEs, especially LFNG, also plays an important role in Notch signaling. Cell-based Notch signaling assays reveal that LFNG elongation facilitates signal induction by DLLs and inhibits signaling from JAGGED (23, 24, 33, 34). LFNG modification of the *O-*fucose on EGF8 and EGF12 is associated with a strong increase in ligand–receptor interaction as well as activation of the Notch pathway, especially in the case of signal induction by DLL1 (23). However, the LFNG modification of *O*-fucose on EGF6 and EGF36, not belonging to the binding domain, decreases the activation of the N1 pathway induced by JAG1. This decrease is not correlated with a decrease in ligand–receptor interaction, which increases in the presence of LFNG (23). The reasons associated with this process are still under investigation.

As mentioned previously, alterations of glycosyltransferases associated with *O-*glycosylation of N1 have been reported in cancers (15, 16). This is notably the case for POFUT1, where amplification, mutation, or upregulation has been found in hepatocellular carcinoma (35), colorectal cancer (36, 37), glioblastoma (38), and squamous cell carcinoma (39). Alteration in FRINGE expression (over, lower, or the absence of expression) has also been found in basal like cancer (40), metastatic melanoma (41), colorectal cancer (42), and claudin-low breast cancer (43). In addition, two *O-*fucosylation site mutations, T^311^P and T^349^P, resulting in loss of *O-*fucose in EGF8 and EGF9, respectively (Fig. 1*A*), have been reported in various cancers. Expression of these mutants compared with N1 WT in human embryonic kidney 293FT cells showed increased proliferation in cells expressing the mutant forms of the receptor (44).

Here, we searched the COSMIC (45) and Biomuta (46) databases for point mutations of the N1 receptor that produce an alteration within the *O-*fucose consensus sequence of EGFs included or close to the ligand-binding domain. After selecting nine mutants meeting these criteria, we compared them with WT N1 using cell-based Notch activation and ligand-binding assays. We also expressed and purified a N1 fragment containing all the EGFs in question (EGF5 to EGF14) in Chinese hamster ovary (CHO) cells in the presence or the absence of LFNG for mass spectral glycoproteomic analysis to evaluate effects of the variants on *O*-fucosylation. Together, these assays allowed us to demonstrate that several mutations resulted in *O-*fucosylation alterations leading to GOF or LOF of N1. Our results showed a correlation between the mutations causing GOF or LOF of N1 observed in our study and the alterations of the Notch pathway found in the corresponding cancers from which they were derived. This work shows that cancer-associated mutations in key areas of the NECD of N1 can cause significant deregulation of the Notch pathway by altering *O-*fucosylation of the receptor, which could be associated with the cancerous process.

## Results

### Identification of point mutations in cancer databases that could affect *O*-fucosylation of N1

According to the Biomuta and COSMIC cancer databases, there are many point mutations affecting the N1 receptor. To limit our research to mutations that could induce a change in *O-*fucosylation likely to affect the Notch pathway, we focused on EGFs in not only the ligand-binding domain (EGF8–12) but also on EGF6 (Fig. 1*A*) whose *O-*fucose elongation inhibits JAG1 activation (23). We found 50 mutations in this region (Table S1). We excluded 29 mutations, which resulted in loss or gain of a cysteine, which would likely cause incorrect folding of the targeted EGF and effects independent of the loss of *O-*glycans. The remaining 21 mutations present an amino acid change in the *O-*fucosylation consensus sequence (C^2^XXXX(S/T)C^3^) that may affect *O-*fucose transfer or elongation. We selected nine of these variants based on their probability to induce an alteration of *O-*fucosylation. These include variants in EGF6 (G^230^R), EGF8 (G^309^R, G^310^R, and T^311^P), EGF9 (G^347^S and T^349^P), and EGF12 (D^464^N and A^465^T) (Fig. 1*B*). The final mutant, EGF10 N^386^T, creates a neo *O-*fucosylation site absent in the WT protein (Fig. 1*B*). These mutations are associated with cancers where N1 had been identified to play either an oncogenic or a tumor suppressor role. For example, the squamous cell carcinoma variants (G^310^R, T^311^P, G^347^S, T^349^P, and A^465^T) are associated with a Notch LOF whereas in acute lymphoblastic T-cell leukemia variants (T^311^P, T^349^P, and N^386^T) are associated with a GOF (4, 5). Interestingly, some mutations are present in different types of cancer where Notch is associated with both GOF or LOF (T^311^P and T^349^P) (Table S2).

### EGF6 G^230^R: a mutation with a limited effect

**Figure 2:**
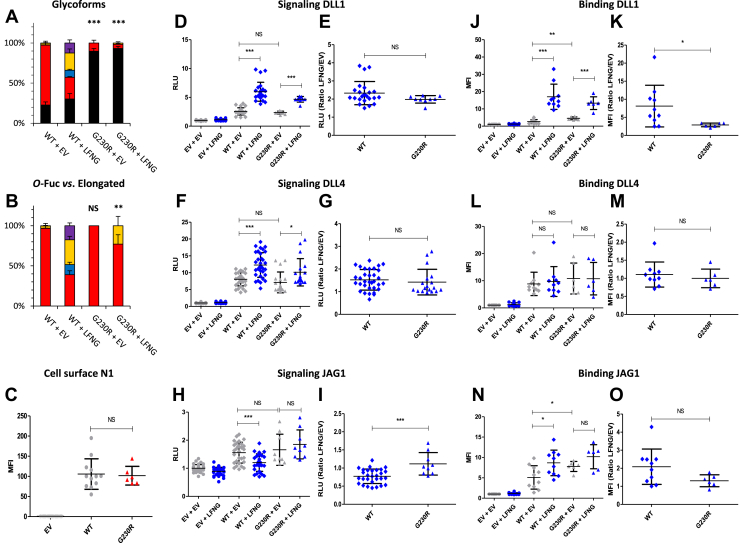
**Effect of mutation G**^**230**^**R on EGF6 *O*-fucosylation and Notch signaling.** CHO cells were cotransfected with plasmids encoding empty vector (EV) or full-length human N1 (WT or G^230^R mutant) and EV or LFNG. *A* and *B*, MS analysis. Statistical tests were performed between N1 WT + EV and G^230^R + EV or N1 WT + LFNG and G^230^R + LFNG. *C*, cell surface N1 quantification. *D*–*I*, cell-based coculture N1 activation assays. *J*–*O*, Notch ligand-binding assays were performed as described in the Experimental procedures section. *A*, quantification of the percentage of unmodified and *O*-fucosylated peptides. *B*, quantification of the percentage of the different *O*-fucosylated peptides (unmodified peptide excluded). Unmodified peptide (*black*), modified by a monosaccharide *O*-fucose (*red*), *O*-fucose + GlcNAc (*blue*), *O*-fucose + GlcNAc + galactose (*yellow*), and *O*-fucose + GlcNAc + galactose + sialic acid (*purple*). The data used to generate the EICs are available in Tables S3–S6. *C*, mean fluorescent intensity for Notch cell surface N1 expression is shown. *D*–*I*, N1 activation assays. Relative luciferase units (RLUs) normalized to EV + EV for coculture N1 activation assays using L-cell or OP9 stably overexpressed DLL1 (*D* and *E*), DLL4 (*F* and *G*), or JAG1 (*H* and *I*) are shown. The effect of LFNG was analyzed by calculating the ratio “LFNG/EV” for DLL1 (*E*), DLL4 (*G*), and JAG1 (*I*). *J*–*O*, N1 ligand–binding assays. Mean fluorescent intensity for Notch ligand–binding assays using DLL1-Fc (*J* and *K*), DLL4-Fc (*L* and *M*), JAG1-Fc (*N* and *O*) is shown. The effect of LFNG was analyzed by calculating the ratio “LFNG/EV” for DLL1 (*K*), DLL4 (*M*), and JAG1 (*O*). Note: The negative (EV, EV + EV, and EV + LFNG) and positive (WT, WT + EV, and WT + LFNG) control data are replotted for all assays (cell surface N1, signaling DLL1, signaling DLL4, signaling JAG1, binding DLL1, binding DLL4, binding JAG1) in Figure 2, Figure 3, Figure 4, Figure 5, Figure 6, Figure 7, Figure 8, Figure 9, Figure 10 to allow comparison with each mutant to WT. CHO, Chinese hamster ovary; DLL, Delta-like ligand; EGF, epidermal growth factor; EIC, extracted ion chromatogram; JAG, JAGGED; LFNG, LUNATIC FRINGE; MS, mass spectrometry; N1, NOTCH1.

The G^230^R mutation located two residues before the *O-*fucosylation site (Fig. 1*B*) changes a residue with no side chain, glycine, to a residue with bulky positively charged side chain, arginine. Although many residues can be found at this position in *O-*fucosylated EGFs, glycine is the most frequent, whereas arginine is very rarely present (47). Prior work has shown that a large residue at this position can lead to a steric clash with POFUT1, resulting in a decrease in affinity of the enzyme for the target EGF (47). Thus, this mutation could decrease *O-*fucosylation and/or affect LFNG elongation of EGF6, which would result in an alteration of Notch signaling. Although this EGF is not localized in the ligand-binding domain, it has been shown that modification of the *O*-fucose on EGF6 by LFNG inhibits induction of N1 signaling by JAG1 (23). Therefore, a change in *O-*fucosylation could have consequences on the ability of JAG1 to activate N1. Our mass spectrometry (MS) results showed that the G^230^R mutation led to a strong decrease in *O-*fucosylation on EGF6 (Fig. 2*A*). This was associated with a statistically significant decrease in the ratio of elongated *O-*fucose in the presence of LFNG (Fig. 2*B*). These *O*-fucosylation changes had no impact on the cell surface receptor expression since comparison of the amount of receptor between WT and G^230^R revealed no difference (Fig. 2*C*). Comparison of WT N1 and the G^230^R mutant in the absence of LFNG revealed no difference in Notch signaling induced by DLL1, DLL4, or JAG1 (Fig. 2, *D*, *F*, and *H*) although this mutant increased receptor interaction with DLL1 and JAG1 slightly (Fig. 2, *J* and *N*). The presence of LFNG induced an increase of WT N1 activation by DLL1 and DLL4 (Fig. 2, *D* and *F*) but a decrease for JAG1 (Fig. 2*H*). Ligand–receptor interaction increased for DLL1 and JAG1 (Fig. 2, *J* and *N*) but remained unchanged for DLL4 (Fig. 2*L*). Similar results were found for the G^230^R mutant regarding signal induction by DLL1 and DLL4 in the presence or the absence of LFNG (Fig. 2, *D* and *F*). Comparison of the LFNG/empty vector (EV) ratio, representative of the effect of LFNG, showed a lower G^230^R–DLL1 interaction compared with WT–DLL1 (Fig. 2*K*). This is most likely because of the increase in G^230^R–DLL1 interaction without LFNG, whereas in the presence of LFNG, this interaction remained similar to the WT–DLL1 interaction (Fig. 2*J*). Interestingly, the decrease of the JAG1-induced signal in the presence of LFNG was no longer present in the G^230^R mutant (Fig. 2*H*), which resulted in a significant increase of the LFNG/EV ratio in this mutant (Fig. 2*I*). No change in the LFNG/EV ratio was observed for G^230^R–JAG1 interaction compared with WT–JAG1 (Fig. 2*O*). The loss of JAG1-induced N1 pathway inhibition in the presence of LFNG because of decreased *O*-fucosylation of EGF6 is consistent with our previous results (23).

### EGF8 G^309^R: a mutation that increased Notch signaling induced by DLL1 and DLL4

**Figure 3:**
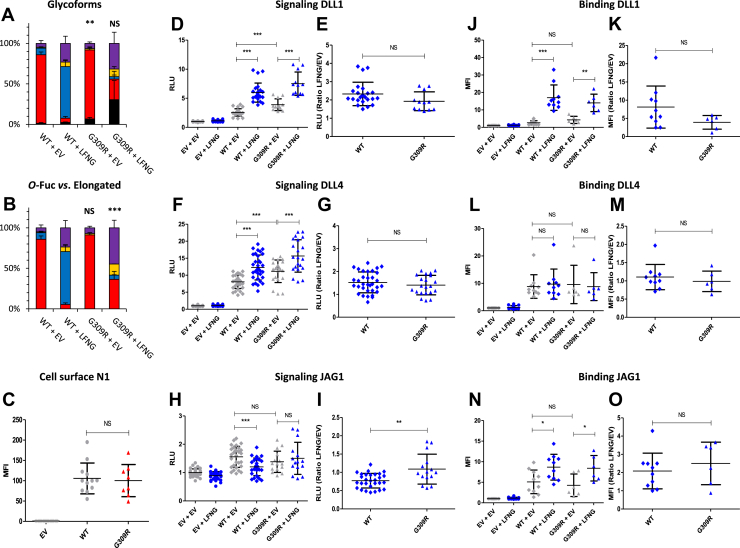
**Effect of mutation G**^**309**^**R on EGF8 *O*-fucosylation and Notch signaling.** CHO cells were cotransfected with plasmids encoding empty vector (EV) or full-length human N1 (WT or G^309^R mutant) and EV or LFNG. *A* and *B*, MS analysis. Statistical tests were performed between N1 WT + EV and G^309^R + EV or N1 WT + LFNG and G^309^R + LFNG. *C*, cell surface N1 quantification. *D*–*I*, cell-based coculture N1 activation assays. *J*–*O*, Notch ligand–binding assays were performed as described in the Experimental procedures section. *A*, quantification of the percentage of unmodified and *O*-fucosylated peptides. *B*, quantification of the percentage of the different *O*-fucosylated peptides (unmodified peptide excluded). Unmodified peptide (*black*), modified by a monosaccharide *O*-fucose (*red*), *O*-fucose + GlcNAc (*blue*), *O*-fucose + GlcNAc + galactose (*yellow*), and *O*-fucose + GlcNAc + galactose + sialic acid (*purple*). The data used to generate the EICs are available in Tables S3, S4, S7, and S8. *C*, mean fluorescent intensity for Notch cell surface N1 expression is shown. *D*–*I*, N1 activation assays. Relative luciferase units (RLUs) normalized to EV + EV for coculture N1 activation assays using L-cell or OP9 stably overexpressed DLL1 (*D* and *E*), DLL4 (*F* and *G*), or JAG1 (*H* and *I*) are shown. The effect of LFNG was analyzed by calculating the ratio “LFNG/EV” for DLL1 (*E*), DLL4 (*G*), and JAG1 (*I*). *J*–*O*, N1 ligand–binding assays. Mean fluorescent intensity for Notch ligand–binding assays using DLL1-Fc (*J* and *K*), DLL4-Fc (*L* and *M*), and JAG1-Fc (*N* and *O*) is shown. The effect of LFNG was analyzed by calculating the ratio “LFNG/EV” for DLL1 (*K*), DLL4 (*M*), and JAG1 (*O*). CHO, Chinese hamster ovary; DLL, Delta-like ligand; EGF, epidermal growth factor; EIC, extracted ion chromatogram; JAG, JAGGED; LFNG, LUNATIC FRINGE; MS, mass spectrometry; N1, NOTCH1.

The G^309^R mutation is in the same position as the G^230^R mutation in EGF6 but targets EGF8 (Fig. 1*B*). Thus, this mutation may impact the ability of POFUT1 to *O*-fucosylate this EGF in the same way as described for EGF6. Analysis of EGF8 *O-*fucosylation by MS showed a small increase in unmodified peptide for the G^309^R mutant, significant only in the absence of LFNG (∼2% and 7% for WT and G^309^R, respectively) (Fig. 3*A*). Elongation by LFNG was less effective in the presence of the G^309^R mutation (∼95% and 65% for WT and G^309^R, respectively) (Fig. 3*B*). This lower elongation for the mutant G^309^R was also visible in the absence of exogenous LFNG (∼15% and 9% for WT and G^309^R, respectively), although this trend was not significant (Fig. 3*B*). Moreover, the elongation profile between the WT and the G^309^R mutant was different, since the major elongated form in WT was the GlcNAc*–*Fuc disaccharide, whereas the majority of *O*-fucose glycans were elongated to tetrasaccharide in the mutant (Fig. 3*B*). No difference in cell surface expression was observed between the WT and the G^309^R mutant (Fig. 3*C*). The EGF8 G^309^R mutation increased Notch pathway activation by DLL1 and DLL4 in the absence of LFNG (Fig. 3, *D* and *F*). The LFNG/EV ratio was similar between WT and G^309^R mutant for both ligands (Fig. 3, *E* and *G*). This increase was not found in our binding assay between the G^309^R mutant and DLL1 or DLL4, whose results were similar to the WT (Fig. 3, *J*–*M*). Regarding signal induction by JAG1, the only difference was the absence of signal decrease in the presence of LFNG for the G^309^R mutant (Fig. 3*H*), which was reflected in a higher LFNG/EV ratio for this mutant compared with WT (Fig. 3*I*). No difference was observed for the N1–JAG1 interaction between the WT and the G^309^R mutant (Fig. 3, *N* and *O*). Since the G^309^R mutation showed a small reduction of *O-*fucosylation and elongation (Fig. 3, *A* and *B*), a negative effect on Notch signaling was expected (23). However, we observed an increase of the signal induced by DLL1 and DLL4 (Fig. 3, *D* and *F*), which could be associated with the switch from the disaccharide form to the tetrasaccharide form observed for the G^309^R mutant. Indeed, the structure of *O*-fucosylglycans has previously been shown to have a role on Notch signaling since it has been reported that the presence of galactose (present only in the trisaccharide and tetrasaccharide forms) enhances the activating effect of the Notch pathway by LFNG (48).

### EGF8 G^310^R and T^311^P: two mutations strongly reducing *O*-fucosylation and Notch signaling

**Figure 4:**
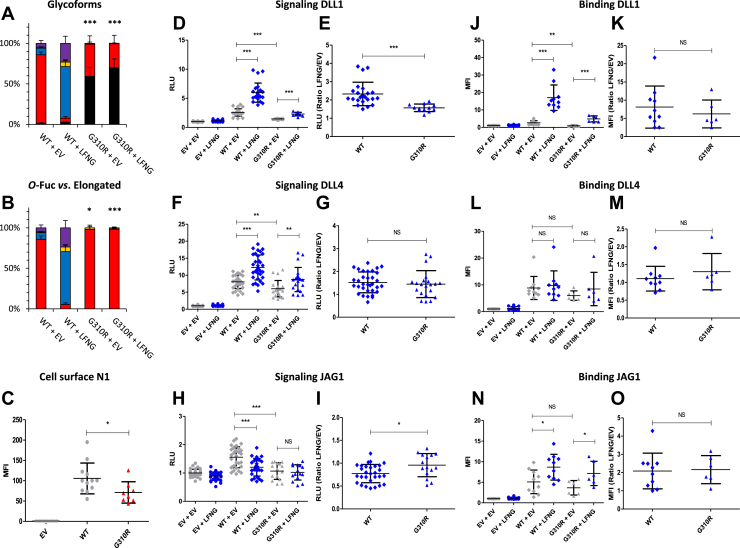
**Effect of mutation G**^**310**^**R on EGF8 *O*-fucosylation and Notch signaling.** CHO cells were cotransfected with plasmids encoding empty vector (EV) or full-length human N1 (WT or G^310^R mutant) and EV or LFNG. *A* and *B*, MS analysis. Statistical tests were performed between N1 WT + EV and G^310^R + EV or N1 WT + LFNG and G^310^R + LFNG. *C*, cell surface N1 quantification. *D*–*I*, cell-based coculture N1 activation assays. *J*–*O*, Notch ligand–binding assays were performed as described in the Experimental procedures section. *A*, quantification of the percentage of unmodified and *O*-fucosylated peptides. *B*, quantification of the percentage of the different *O*-fucosylated peptides (unmodified peptide excluded). Unmodified peptide (*black*), modified by a monosaccharide *O*-fucose (*red*), *O*-fucose + GlcNAc (*blue*), *O*-fucose + GlcNAc + galactose (*yellow*), and *O*-fucose + GlcNAc + galactose + sialic acid (*purple*). The data used to generate the EICs are available in Tables S3, S4, S9, and S10. *C*, mean fluorescent intensity for Notch cell surface N1 expression is shown. *D*–*I*, N1 activation assays. Relative luciferase units (RLUs) normalized to EV + EV for coculture N1 activation assays using L-cell or OP9 stably overexpressed DLL1 (*D* and *E*), DLL4 (*F* and *G*), or JAG1 (*H* and *I*) is shown. The effect of LFNG was analyzed by calculating the ratio “LFNG/EV” for DLL1 (*E*), DLL4 (*G*), and JAG1 (*I*). *J*–*O*, N1 ligand–binding assays. Mean fluorescent intensity for Notch ligand–binding assays using DLL1-Fc (*J* and *K*), DLL4-Fc (*L* and *M*), and JAG1-Fc (*N* and *O*) is shown. The effect of LFNG was analyzed by calculating the ratio “LFNG/EV” for DLL1 (*K*), DLL4 (*M*), and JAG1 (*O*). CHO, Chinese hamster ovary; DLL, Delta-like ligand; EGF, epidermal growth factor; EIC, extracted ion chromatogram; JAG, JAGGED; LFNG, LUNATIC FRINGE; MS, mass spectrometry; N1, NOTCH1.

**Figure 5:**
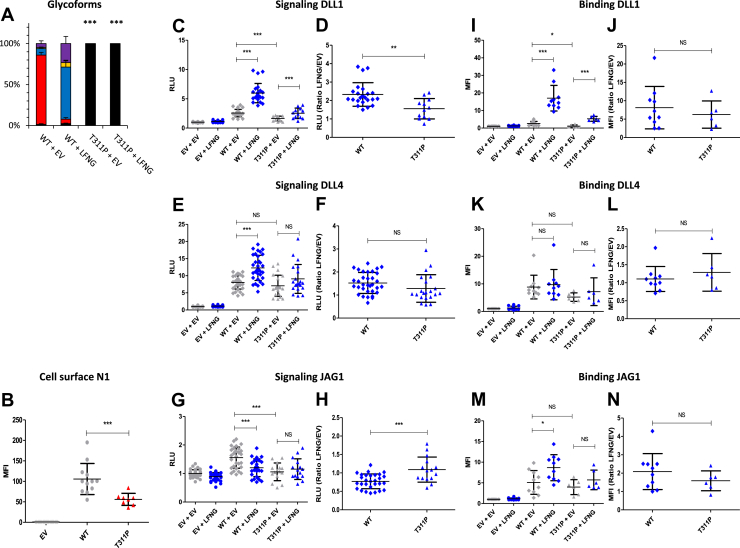
**Effect of mutation T**^**311**^**P on EGF8 *O*-fucosylation and Notch signaling.** CHO cells were cotransfected with plasmids encoding empty vector (EV) or full-length human N1 (WT or T^311^P mutant) and EV or LFNG. *A*, MS analysis. Statistical tests were performed between N1 WT + EV and T^311^P + EV or N1 WT + LFNG and T^311^P + LFNG. *B*, cell surface N1 quantification. *C*–*H*, cell-based coculture N1 activation assays. *I*–*N*, Notch ligand–binding assays were performed as described in the Experimental procedures section. *A*, quantification of the percentage of unmodified and *O*-fucosylated peptides. Unmodified peptide (*black*), modified by a monosaccharide *O*-fucose (*red*), *O*-fucose + GlcNAc (*blue*), *O*-fucose + GlcNAc + galactose (*yellow*), and *O*-fucose + GlcNAc + galactose + sialic acid (*purple*). The data used to generate the EICs are available in Tables S3, S4, S11, and S12. *B*, mean fluorescent intensity for Notch cell surface N1 expression is shown. *C*–*H*, N1 activation assays. Relative luciferase units (RLUs) normalized to EV + EV for coculture N1 activation assays using L-cell or OP9 stably overexpressed DLL1 (*C* and *D*), DLL4 (*E* and *F*), or JAG1 (*G* and *H*) are shown. The effect of LFNG was analyzed by calculating the ratio “LFNG/EV” for DLL1 (*D*), DLL4 (*F*), and JAG1 (*H*). *I*–*N*, N1 ligand–binding assays. Mean fluorescent intensity for Notch ligand–binding assays using DLL1-Fc (*I* and *J*), DLL4-Fc (*K* and *L*), JAG1-Fc (*M* and *N*) is shown. The effect of LFNG was analyzed by calculating the ratio “LFNG/EV” for DLL1 (*J*), DLL4 (*L*), and JAG1 (*N*). CHO, Chinese hamster ovary; DLL, Delta-like ligand; EGF, epidermal growth factor; EIC, extracted ion chromatogram; JAG, JAGGED; LFNG, LUNATIC FRINGE; MS, mass spectrometry; N1, NOTCH1.

The G^310^R and T^311^P mutations affect the residue just before the *O-*fucosylation site and the *O-*fucosylation site itself, respectively (Fig. 1*B*). Interestingly, the G^310^R mutant, which contains the *O-*fucosylation site, showed a large increase in unmodified peptide (∼60%) compared with the WT (∼1%) (Fig. 4*A*). As expected, the loss of the T^311^ *O-*fucosylation site for the T^311^P mutant resulted in a total absence of *O-*fucose (Fig. 5*A*). The G^310^R mutant also strongly reduced *O*-fucose modification by LFNG (Fig. 4, *B*). Both mutants showed a decrease in the amount of N1 on the cell surface (Figs. 4*C* and 5B). Both mutations induced a strong decrease in DLL1- and JAG1-induced signaling (Figs. 4, *D*, *H*, 5, *C*, and *G*) and a smaller decrease in DLL4 signaling for the G^310^R mutant (Fig. 4*F*). Despite a significant increase in DLL1-induced signal in the presence of LFNG for each mutant, this increase was smaller than for WT (Figs. 4*D* and 5C) as evidenced by the lower LFNG/EV ratio observed for these mutants (Figs. 4*E* and 5D). Both mutants showed a similar ligand-binding profile with a decrease of the N1–DLL1 interaction without modification of the LFNG/EV ratio (Figs. 4, *J*, *K*, 5, I, and *J*). No difference between mutants and WT was found for the N1–DLL4 or N1–JAG1 interaction (Figs. 4, *L*–*O* and 5, *K*–*N*). These results correlate with our previous study, where the use of murine N1 with a mutated EGF8 *O-*fucosylation site demonstrated the important role of this *O-*fucose in the proper function of the Notch pathway (23).

### EGF9 G^347^S and T^349^P: two mutations reducing Notch signaling to different degrees

**Figure 6:**
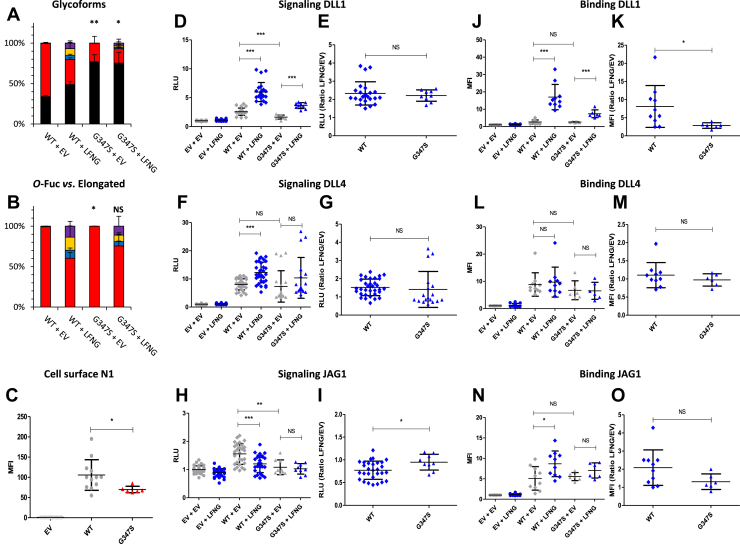
**Effect of mutation G**^**347**^**S on EGF9 *O*-fucosylation and Notch signaling.** CHO cells were cotransfected with plasmids encoding empty vector (EV) or full-length human N1 (WT or G^347^S mutant) and EV or LFNG. *A* and *B*, MS analysis. Statistical tests were performed between N1 WT + EV and G^347^S + EV or N1 WT + LFNG and G^347^S + LFNG. *C*, cell surface N1 quantification. *D*–*I*, cell-based coculture N1 activation assays. *J*–*O*, Notch ligand–binding assays were performed as described in the Experimental procedures section. *A*, quantification of the percentage of unmodified and *O*-fucosylated peptides. *B*, quantification of the percentage of the different *O*-fucosylated peptides (unmodified peptide excluded). Unmodified peptide (*black*), modified by a monosaccharide *O*-fucose (*red*), *O*-fucose + GlcNAc (*blue*), *O*-fucose + GlcNAc + galactose (*yellow*), and *O*-fucose + GlcNAc + galactose + sialic acid (*purple*). The data used to generate the EICs are available in Tables S3, S4, S13, and S14. *C*, mean fluorescent intensity for Notch cell surface N1 expression is shown. *D*–*I*, N1 activation assays. Relative luciferase units (RLUs) normalized to EV + EV for coculture N1 activation assays using L-cell or OP9 stably overexpressed DLL1 (*D* and *E*), DLL4 (*F* and *G*), or JAG1 (*H* and *I*) are shown. The effect of LFNG was analyzed by calculating the ratio “LFNG/EV” for DLL1 (*E*), DLL4 (*G*), and JAG1 (*I*). *J*–*O*, N1 ligand–binding assays. Mean fluorescence intensity for Notch ligand–binding assays using DLL1-Fc (*J* and *K*), DLL4-Fc (*L* and *M*), and JAG1-Fc (*N* and *O*) is shown. The effect of LFNG was analyzed by calculating the ratio “LFNG/EV” for DLL1 (*K*), DLL4 (*M*), and JAG1 (*O*). CHO, Chinese hamster ovary; DLL, Delta-like ligand; EGF, epidermal growth factor; EIC, extracted ion chromatogram; JAG, JAGGED; LFNG, LUNATIC FRINGE; MS, mass spectrometry; N1, NOTCH1.

Like EGF6 G^230^R and EGF8 G^309^R, the G^347^S mutant is located two residues before the *O-*fucosylation site (Fig. 1*B*). Although this change seems less drastic than the glycine to arginine switch, an alteration of the *O-*fucosylation is possible. MS analysis showed that the G^347^S mutation resulted in a decrease in the proportion of *O-*fucosylated peptide compared with WT (∼65% and 25% for WT and G^347^S, respectively) (Fig. 6*A*). The proportion of extended *O-*fucose in the presence of LFNG also decreased, although this trend was not significant (Fig. 6*B*). This mutation reduced the expression of N1 at the cell surface (Fig. 6*C*). The G^347^S mutation also resulted in a decrease in DLL1-induced signaling (Fig. 6*D*) without change in the LFNG/EV ratio (Fig. 6*E*). The N1–DLL1 interaction was not affected in the absence of LFNG (Fig. 6*J*), but we observed a decrease in the LFNG/EV ratio for the G^347^S mutant compared with WT (Fig. 6*K*). The signal and interaction associated with DLL4 was similar to WT (Fig. 6, *F*, *G*, *L*, and *M*). JAG1-induced signaling was also decreased for the G^347^S mutant (Fig. 6*H*). We also observed a loss of the JAG1-induced decrease because of LFNG (Fig. 6*H*) resulting in a higher LFNG/EV ratio compared with WT (Fig. 6*I*). The interaction between JAG1 and N1 was not affected (Fig. 6, *N* and *O*).

**Figure 7:**
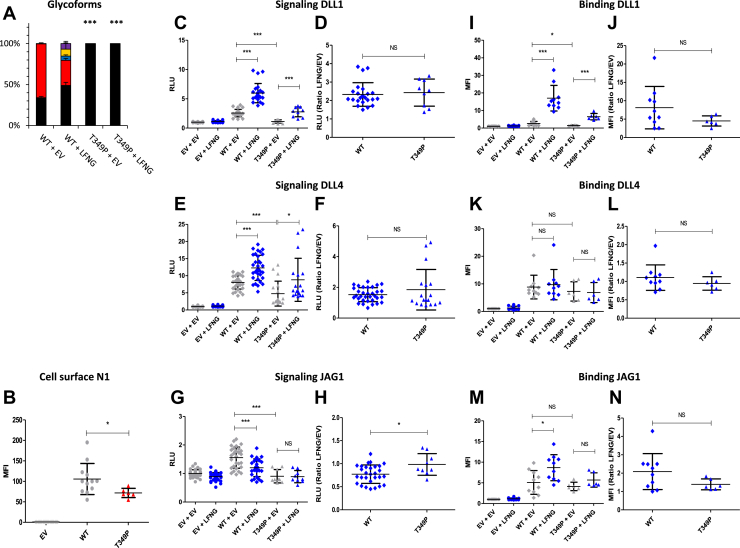
**Effect of mutation T**^**349**^**P on EGF9 *O*-fucosylation and Notch signaling.** CHO cells were cotransfected with plasmids encoding empty vector (EV) or full-length human N1 (WT or T^349^P mutant) and EV or LFNG. *A*, MS analysis. Statistical tests were performed between N1 WT + EV and T^349^P + EV or N1 WT + LFNG and T^349^P + LFNG. *B*, cell surface N1 quantification. *C*–*H*, cell-based coculture N1 activation assays. *I*–*N*, Notch ligand–binding assays were performed as described in the Experimental procedures section. *A*, quantification of the percentage of unmodified and *O*-fucosylated peptides. Unmodified peptide (*black*), modified by a monosaccharide *O*-fucose (*red*), *O*-fucose + GlcNAc (*blue*), *O*-fucose + GlcNAc + galactose (*yellow*), and *O*-fucose + GlcNAc + galactose + sialic acid (*purple*). The data used to generate the EICs are available in Tables S3, S4, S15, and S16. *B*, mean fluorescent intensity for Notch cell surface N1 expression is shown. *C*–*H*, N1 activation assays. Relative luciferase units (RLUs) normalized to EV + EV for coculture N1 activation assays using L-cell or OP9 stably overexpressed DLL1 (*C* and *D*), DLL4 (*E* and *F*), or JAG1 (*G* and *H*) is shown. The effect of LFNG was analyzed by calculating the ratio “LFNG/EV” for DLL1 (*D*), DLL4 (*F*), and JAG1 (*H*). *I*–*N*, N1 ligand–binding assays. Mean fluorescent intensity for Notch ligand–binding assays using DLL1-Fc (*I* and *J*), DLL4-Fc (*K* and *L*), and JAG1-Fc (*M* and *N*) is shown. The effect of LFNG was analyzed by calculating the ratio “LFNG/EV” for DLL1 (*J*), DLL4 (*L*), and JAG1 (*N*). CHO, Chinese hamster ovary; DLL, Delta-like ligand; EGF, epidermal growth factor; EIC, extracted ion chromatogram; JAG, JAGGED; LFNG, LUNATIC FRINGE; MS, mass spectrometry; N1, NOTCH1.

The T^349^P mutation consists of a loss of the *O-*fucosylation site of EGF9 (Fig. 1*B*). The loss of *O-*fucosylation was found by our MS analysis (Fig. 7*A*) and was associated by a decrease in the expression of the N1 receptor at the cell surface (Fig. 7*B*). These effects were similar to those of the G^347^S mutant (Fig. 7, *C*–*N*) but were more drastic with a significant decrease of N1–DLL1 signaling (Fig. 7*C*) and binding (Fig. 7*I*) and a decrease of the DLL4-induced signal (Fig. 7*E*). As with EGF8, loss of *O-*fucose of EGF9 reduced cell surface expression of murine N1 (33). It is therefore not surprising to see the same effect for the T^349^P mutant deleting the *O-*fucosylation site, or for the G^347^S mutant, both resulting in a strong decrease of *O-*fucose. Unlike our previous studies where the loss of *O-*fucose from EGF9 had little effect on the activation of the Notch pathway (23, 33), here, we observed a strong decrease. However, previous studies were performed by overexpressing the murine N1 receptor in NIH3T3 or human embryonic kidney 293T cell lines. It is possible that the human protein behaves differently from the murine protein. Moreover, as shown in our previous study, N1-induced signaling differs from one cell line to another (24). This probably depends on the endogenous levels of Notch receptors as well as the glycosyltransferases POFUT1 and FRINGE. Thus, the overexpression of human N1 in a CHO cell line alone could explain these differences.

### EGF10 N^386^T: a mutation that resulted in a neo *O*-fucosylation site leading to hyperactivation of the Notch pathway

**Figure 8:**
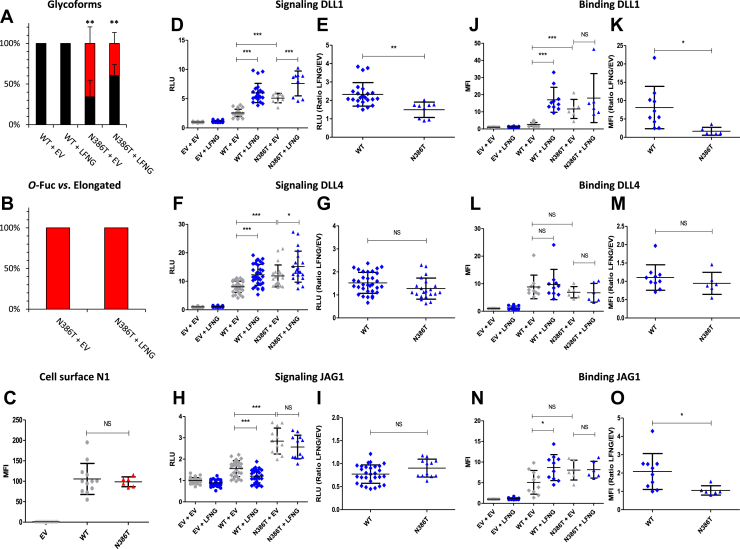
**Effect of mutation N**^**386**^**T on EGF10 *O*-fucosylation and Notch signaling.** CHO cells were cotransfected with plasmids encoding empty vector (EV) or full-length human N1 (WT or N^386^T mutant) and EV or LFNG. *A* and *B*, MS analysis. Statistical tests were performed between N1 WT + EV and N^386^T + EV or N1 WT + LFNG and N^386^T + LFNG. *C*, cell surface N1 quantification. *D*–*I*, cell-based coculture N1 activation assays. *J*–*O*, Notch ligand–binding assays were performed as described in the Experimental procedures section. *A*, quantification of the percentage of unmodified and *O*-fucosylated peptides. *B*, quantification of the percentage of the different *O*-fucosylated peptides (unmodified peptide excluded). Unmodified peptide (*black*), modified by a monosaccharide *O*-fucose (*red*), *O*-fucose + GlcNAc (*blue*), *O*-fucose + GlcNAc + galactose (*yellow*), and *O*-fucose + GlcNAc + galactose + sialic acid (*purple*). The data used to generate the EICs are available in Tables S3, S4, S17, and S18. *C*, mean fluorescent intensity for Notch cell surface N1 expression is shown. *D*–*I*, N1 activation assays. Relative luciferase units (RLUs) normalized to EV + EV for coculture N1 activation assays using L-cell or OP9 stably overexpressed DLL1 (*D* and *E*), DLL4 (*F* and *G*), or JAG1 (*H* and *I*) are shown. The effect of LFNG was analyzed by calculating the ratio “LFNG/EV” for DLL1 (*E*), DLL4 (*G*), and JAG1 (*I*). *J*–*O*, N1 ligand–binding assays. Mean fluorescent intensity for Notch ligand–binding assays using DLL1-Fc (*J* and *K*), DLL4-Fc (*L* and *M*), and JAG1-Fc (*N* and *O*) is shown. The effect of LFNG was analyzed by calculating the ratio “LFNG/EV” for DLL1 (*K*), DLL4 (*M*), and JAG1 (*O*). CHO, Chinese hamster ovary; DLL, Delta-like ligand; EGF, epidermal growth factor; EIC, extracted ion chromatogram; JAG, JAGGED; LFNG, LUNATIC FRINGE; MS, mass spectrometry; N1, NOTCH1.

The N^386^T mutation introduced a neo *O-*fucosylation site in the ligand-binding domain of the N1 receptor (Fig. 1*B*). Our MS results showed that this new *O-*fucosylation site was about 50% occupied (Fig. 8*A*) and that LFNG did not modify this new *O-*fucose (Fig. 8, *A* and *B*). The cell surface expression of the N^386^T mutant was similar to WT (Fig. 8*C*). This mutation resulted in a significant increase in Notch signaling induced by all ligands (Fig. 8, *D*, *F*, and *H*). Interestingly, we observed a decrease in the LFNG/EV ratio for DLL1 likely because of the increased signal in the absence of LFNG (Fig. 8*E*). The N1–DLL1 interaction followed the signaling trend with an increase in interaction for the N^386^T mutant (Fig. 8*J*) associated with a decrease in LFNG/EV ratio (Fig. 8*K*). No difference was found for the N1–DLL4 interaction between the N^386^T mutant and the WT (Fig. 8, *L* and *M*). Although the increase in N1–JAG1 interaction of the mutant in the absence of LFNG was not statistically significant, an increased trend was observed (Fig. 8*N*). This was associated with a loss of the increased binding caused by LFNG for the N^386^T mutant (Fig. 8*N*), resulting in a decrease of the LFNG/EV ratio (Fig. 8*O*). Thus, the addition of an *O-*fucose on EGF10 hyperactivated the N1 receptor. However, this hyperactivation could be caused by a change in the stoichiometry of *O-*fucosylation on other EGFs and not the *O-*fucose of EGF10 itself. Comparison of the *O-*fucosylation of EGF6, 8, 9, and 12 between the WT and the N^386^T mutant showed no difference, demonstrating that this effect was due to changes of the *O-*fucose on EGF10 (Fig. S1). To strengthen this hypothesis, an automated homology model was generated using the cocrystal structure between rat N1 and JAG1 (32). This model shows the EGF10 N^386^T *O-*fucose in proximity of the JAG1 ligand, suggesting the possible involvement of this *O-*fucose in the receptor–ligand interaction facilitating the activation of the Notch pathway (Fig. S2).

### EGF12 D^464^N: a mutation that increased *O*-fucosylation of EGF12 but reduced Notch signaling

**Figure 9:**
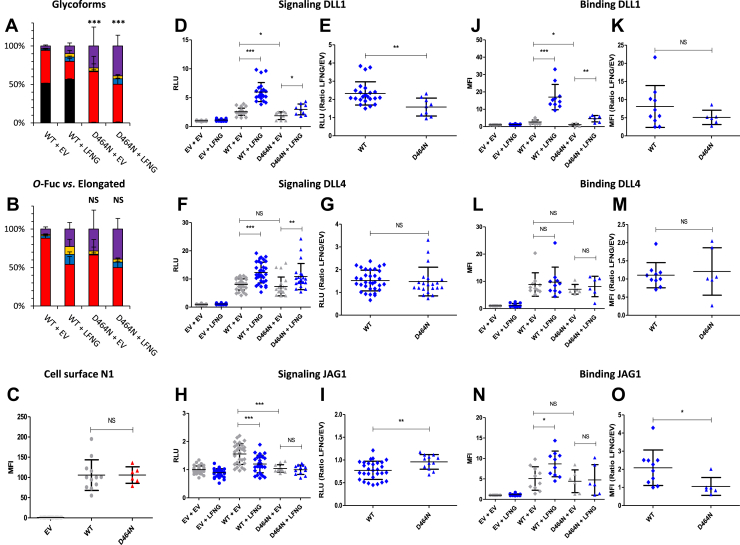
**Effect of mutation D**^**464**^**N on EGF12 *O*-fucosylation and Notch signaling.** CHO cells were cotransfected with plasmids encoding empty vector (EV) or full-length human N1 (WT or D^464^N mutant) and EV or LFNG. *A* and *B*, MS analysis. Statistical tests were performed between N1 WT + EV and D^464^N + EV or N1 WT + LFNG and D^464^N + LFNG. *C*, cell surface N1 quantification. *D*–*I*, cell-based coculture N1 activation assays. *J*–*O*, Notch ligand–binding assays were performed as described in the Experimental procedures section. *A*, quantification of the percentage of unmodified and *O*-fucosylated peptides. *B*, quantification of the percentage of the different *O*-fucosylated peptides (unmodified peptide excluded). Unmodified peptide (*black*), modified by a monosaccharide *O*-fucose (*red*), *O*-fucose + GlcNAc (*blue*), *O*-fucose + GlcNAc + galactose (*yellow*), and *O*-fucose + GlcNAc + galactose + sialic acid (*purple*). The data used to generate the EICs are available in Tables S3, S4, S19, and S20. *C*, mean fluorescent intensity for Notch cell surface N1 expression is shown. *D*–*I*, N1 activation assays. Relative luciferase units (RLUs) normalized to EV + EV for coculture N1 activation assays using L-cell or OP9 stably overexpressed DLL1 (*D* and *E*), DLL4 (*F* and *G*), or JAG1 (*H* and *I*) are shown. The effect of LFNG was analyzed by calculating the ratio “LFNG/EV” for DLL1 (*E*), DLL4 (*G*), and JAG1 (*I*). *J*–*O*, N1 ligand–binding assays. Mean fluorescent intensity for Notch ligand–binding assays using DLL1-Fc (*J* and *K*), DLL4-Fc (*L* and *M*), and JAG1-Fc (*N* and *O*) is shown. The effect of LFNG was analyzed by calculating the ratio “LFNG/EV” for DLL1 (*K*), DLL4 (*M*), and JAG1 (*O*). CHO, Chinese hamster ovary; DLL, Delta-like ligand; EGF, epidermal growth factor; EIC, extracted ion chromatogram; JAG, JAGGED; LFNG, LUNATIC FRINGE; MS, mass spectrometry; N1, NOTCH1.

The D^464^N mutation is located two residues before the *O-*fucosylation site of EGF12 (Fig. 1*B*). The aspartate normally present at this position induces a steric clash with a POFUT1 residue reducing the affinity of the enzyme for this EGF12. Mutation of this aspartate to glycine showed a strong improvement in the affinity of POFUT1 for EGF12 (47). The presence of an asparagine for this mutant would be predicted to have limited effect unless elimination of the negative charge from the aspartate is a significant component of the effect. Interestingly, the D^464^N mutation significantly increased *O-*fucosylation of EGF12 with the percentage of unmodified peptide reduced from ∼50% for the WT to <1% for the D^464^N mutant (Fig. 9*A*). Despite a tendency to increase, the percentage of elongated *O-*fucose for the D^464^N mutant remained similar to WT in the presence or the absence of LFNG (Fig. 9*B*). The presence of N1 at the cell surface was not affected by this mutation (Fig. 9*C*). The D^464^N mutation caused a decrease in Notch pathway induction by DLL1 and JAG1 (Fig. 9, *D* and *H*, respectively) associated with a decrease or an increase in the LFNG/EV ratio depending on the effect of LFNG (enhancer with DLL1 or inhibitor with JAG1) (Fig. 9, E and I, respectively). DLL4-induced signaling was not affected by this mutation (Fig. 9, *F* and *G*). The DLL1 interaction was also slightly affected with a decrease of binding to the mutant (Fig. 9*J*). A loss of the LFNG-induced increase for JAG1 binding was also observed (Fig. 9*N*) associated with a decrease in the LFNG/EV ratio (Fig. 9*O*). The fact that the mutation caused a decrease in Notch activation by DLL1 and JAG1 was surprising since *O*-fucosylation of EGF12 was increased. This change in stoichiometry could have a negative effect on *O-*fucosylation of another EGF. We analyzed the EGF8 site, where a decrease in *O-*fucosylation could have explained our results. However, no difference in EGF8 *O-*fucosylation between the D^464^N mutant and the WT was observed (Fig. S3). There is also an *O*-glucose modification on the EGF12 peptide in addition to the *O*-fucose (Fig. S6*E*). The *O*-glucose was in the trisaccharide form on nearly 90% of all peptide spectral matches for the EGF12 peptide in WT and D^464^N mutant (Tables S3, S4, S19, and S20), suggesting that the mutation is not affecting *O*-glucose modifications. Another possibility would be that the aspartate residue is necessary for the ligand–receptor interaction and that its replacement by an asparagine weakens this interaction. Using automated homology model and MatchMaker of CHIMERA, we created a model for D^464^N mutant and superimposed it on the cocrystal structures of N1–JAG1 or N1–DLL4 (Fig. S4). Comparison of the models with the reference structure does not suggest that the aspartate residue is directly involved in the interaction with JAG1 or DLL4. It is therefore unlikely that its replacement by an asparagine would result in a decrease in interaction responsible for our results (Fig. S4).

### EGF12 A^465^T: a mutation that reduced LFNG-mediated elongation and significantly reduced induction of the Notch pathway

**Figure 10:**
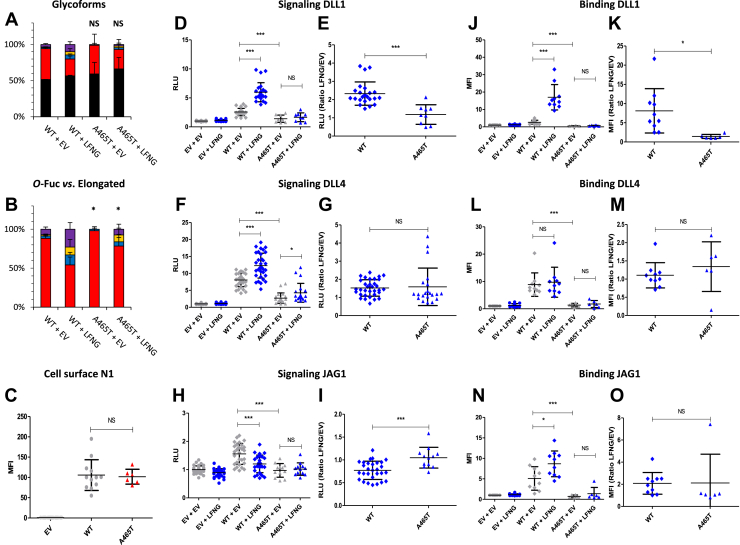
**Effect of mutation A**^**465**^**T on EGF12 *O*-fucosylation and Notch signaling.** CHO cells were cotransfected with plasmids encoding empty vector (EV) or full-length human N1 (WT or A^465^T mutant) and EV or LFNG. *A* and *B*, MS analysis. Statistical tests were performed between N1 WT + EV and A^465^T + EV or N1 WT + LFNG and A^465^T + LFNG. *C*, cell surface N1 quantification. *D*–*I*, cell-based coculture N1 activation assays. *J*–*O*, Notch ligand–binding assays were performed as described in the Experimental procedures section. *A*, quantification of the percentage of unmodified and *O*-fucosylated peptides. *B*, quantification of the percentage of the different *O*-fucosylated peptides (unmodified peptide excluded). Unmodified peptide (*black*), modified by a monosaccharide *O*-fucose (*red*), *O*-fucose + GlcNAc (*blue*), *O*-fucose + GlcNAc + galactose (*yellow*), and *O*-fucose + GlcNAc + galactose + sialic acid (*purple*). The data used to generate the EICs are available in Tables S3, S4, S21, and S22. *C*, Mean fluorescent intensity for Notch cell surface N1 expression is shown. *D*–*I*, N1 activation assays. Relative luciferase units (RLUs) normalized to EV + EV for coculture N1 activation assays using L-cell or OP9 stably overexpressed DLL1 (*D* and *E*), DLL4 (*F* and *G*), or JAG1 (*H* and *I*) are shown. The effect of LFNG was analyzed by calculating the ratio “LFNG/EV” for DLL1 (*E*), DLL4 (*G*), and JAG1 (*I*). *J*–*O*, N1 ligand–binding assays. Mean fluorescent intensity for Notch ligand–binding assays using DLL1-Fc (*J* and *K*), DLL4-Fc (*L* and *M*), and JAG1-Fc (*N* and *O*) is shown. The effect of LFNG was analyzed by calculating the ratio “LFNG/EV” for DLL1 (*K*), DLL4 (*M*), and JAG1 (*O*). CHO, Chinese hamster ovary; DLL, Delta-like ligand; EGF, epidermal growth factor; EIC, extracted ion chromatogram; JAG, JAGGED; LFNG, LUNATIC FRINGE; MS, mass spectrometry; N1, NOTCH1.

The A^465^T mutation is located one residue before the *O-*fucosylation site (Fig. 1*B*). At this position, residues with a small or nonexistent side chain such as glycine, alanine, and more rarely serine are found in POFUT1-modified EGFs (47). Thus, the presence of a threonine could disrupt the *O-*fucosylation of EGF12. Surprisingly, no difference in the proportion of *O-*fucosylation of EGF12 was observed between the WT and A^465^T mutant (Fig. 10*A*). However, the ratio of LFNG-mediated elongation *O-*fucose was lower in the mutant compared with the WT. In the absence of LFNG, ∼12% elongation was observed for the WT and ∼2% for the A^465^T mutant, whereas with LFNG, ∼45% was observed for the WT and ∼22% for the mutant (Fig. 10*B*). No difference between WT and A^465^T mutant was found for cell surface expression (Fig. 10*C*). This mutant induced a drastic decrease in signal induction and binding by all ligands (Fig 10, *D*, *F*, *H*, *J*, *L*, and *N*). Moreover, it strongly affected the ability of LFNG to enhance DLL1-induced signaling and binding (Fig. 10, *D*, *E*, *J*, and *K*) and to inhibit JAG1-induced signaling (Fig. 10, H and I). We created an automated homology model and MatchMaker of CHIMERA for the A^465^T mutant to check if the residue at position 465 is involved in the interaction of N1 with its ligands and superimposed it on the cocrystal structures of N1–JAG1 or N1–DLL4 (Fig. S5). The position of A^465^ or its mutated counterpart T^465^ does not appear to be directly involved in the interaction with its ligands DLL4 and JAG1.

## Discussion

**Figure 11:**
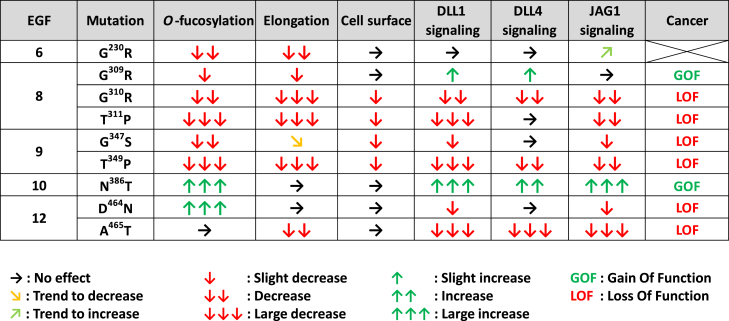
**Summary of the effects for each mutation and possible roles in cancer**.

In this study, we showed that point mutations found in a variety of cancers can affect the *O-*fucosylation of key EGFs of N1 and deregulate activation of the Notch pathway. Among the nine selected mutations, two led to an N1 GOF (G^309^R and N^386^T), six to an N1 LOF (G^310^R, T^311^P, G^347^S, T^349^P, D^464^N, and A^465^T), and one had little or no effect (G^230^R). Figure 11 summarizes the results obtained for each mutant in comparison with WT for N1 cell surface expression, activation of the N1 pathway by its ligands, and EGF *O-*fucosylation associated with each mutation. Our results show that most of the effects of these mutations on N1 activation are consistent with the role of N1 in the cancers from which they were derived.

The T^311^P (EGF8) and T^349^P (EGF9) caused a complete loss of *O*-fucosylation of these EGFs leading to a decrease in N1 cell surface expression and a strong decrease in Notch activation by DLL1, DLL4, and JAG1 (Fig. 11). Thus, our results showed that these mutations induce an LOF of N1. This is consistent with the essential role of *O-*fucose on EGF8 in the activation of the Notch pathway (23, 27, 28, 30). The *O-*fucose of EGF9 seems less essential, but its loss has been associated with decreased receptor expression at the cell surface (23, 33). Interestingly, LOF of the Notch pathway is known to be prevalent in certain cancers (4), including squamous cell carcinoma of the skin (6), head and neck (7), and esophagus (8). Both the T^311^P and T^349^P mutations have been found in squamous cell carcinoma of head and neck with high frequency (23 and 20, respectively) (Table S2). Thus, the LOF associated with the T^311^P and T^349^P mutations is consistent with the role of N1 in squamous cell carcinoma.

The G^310^R (EGF8) and G^347^S (EGF9) showed very similar results to T^311^P (EGF8) and T^349^P (EGF9) mutations. Indeed, although the *O*-fucosylation site is still present, a strong decrease of *O*-fucosylation was observed. This led to a strong N1 LOF, similar to T^311^P (EGF8) and T^349^P (EGF9) mutants (Fig. 11). The G^310^R (EGF8) and G^347^S (EGF9) have also been found in squamous cell carcinoma of head and neck and in other squamous cell carcinomas for the G^310^R mutation (Table S2), consistent with N1 LOF in squamous cell carcinomas.

The N^386^T (EGF10) mutation created a neo *O-*fucosylation site within the ligand-binding domain of N1 (Fig. 1). This caused a hyperactivation of the N1 induced by DLL1, DLL4, and JAG1 (Fig. 11). Our model showed this new *O-*fucose in close proximity to the JAG1 ligand and could therefore be involved in enhancing ligand–receptor interactions (Fig. S2). The *O-*fucose of EGF10 might also play a stabilizing role for the N1 receptor, which could facilitate the conformational change necessary for its activation during the exertion of the ligand's pulling force on N1. This N1 mutation inducing a GOF has only been found in T-cell acute lymphoblastic leukemia (Table S2), which is consistent with other studies associating N1 GOFs with this cancer (4, 11, 12).

The G^309^R (EGF8) mutation caused a GOF by allowing hyperactivation of N1 induced by DLL1 and DLL4 (Fig. 11). However, the reason for this hyperactivation remains unclear. The mutation caused a loss of the disaccharide form of *O*-fucose (GlcNAcβ1,3Fucose) in the absence or the presence of LFNG (Fig. 3*B*). Prior studies have explored how elongation of *O-*fucose beyond the disaccharide affects N1 activity (48). Additional studies focusing on EGF8 elongation may provide additional clarification of these results. The G^309^R mutation has been found only once in stomach cancer (Table S2). Unfortunately, no more precise information on the nature of this cancer allows us to make a relation between the known role of Notch in this cancer and the GOF observed in our study.

The D^464^N (EGF12) mutation was associated with an increased *O-*fucosylation of EGF12 (Fig. 11). This confirmed that the relatively low abundance of *O-*fucosylation of EGF12 is due to the presence of the aspartate residue at position C^2^ + 2, which is known to induce a steric clash with tyrosine 78 of POFUT1 (47). However, the effect of increasing the *O-*fucose stoichiometry did not have the anticipated effect on N1 activity. Indeed, it is known that the loss of *O-*fucose from EGF12 leads to a reduction of Notch signaling (23, 29). Thus, the increase in *O-*fucosylation without affecting the proportion of LFNG elongation would suggest an overactivation of the pathway. However, our results showed the opposite effect: a decrease in the activation of N1 by DLL1 and JAG1 (Fig. 11). The mechanisms underlying these results remain unclear. It is possible that the role of *O-*fucose on EGF12 is clearly dependent on its stoichiometry and that too much or too little *O-*fucose alters signaling. The mutation may slightly modify the structure of EGF12, which would slightly shift the position of the *O-*fucosylated threonine side chain. Since the *O-*fucose of EGF12 interacts directly with its ligands (31, 32), even a slight change in its position could alter this interaction and decrease their ability to induce Notch signaling. In any case, this mutation leads to an N1 LOF. Unfortunately, the details of the cancers where these mutations have been found are not precise enough to allow us to correlate our results with any known deregulation of the Notch pathway (Table S2).

The A^465^T (EGF12) mutation resulted in a drastic decrease of N1 activation by all ligands. *O-*fucosylation was not modified, but the elongation of *O-*fucose was lower in the mutant with or without LFNG (Figs. 10 and 11). This suggests that even a small amount of *O*-fucose extension on EGF12 by a Fringe could have a large effect on N1 activation. Alternatively, as with the D^464^N mutation, A^465^T could lead to a conformational change in the side chain of the *O-*fucosylated threonine leading to a reduced interaction with N1 ligands. It is interesting to note that the A^465^T mutation induced a larger decrease in N1 activation or ligand binding compared with the D^464^N mutant (Fig. 11). Being closer to the *O-*fucosylated threonine, A^465^T could generate a larger displacement of the *O*-fucosylated threonine compared with the D^464^N mutant. This would lead to a stronger decrease of the interaction and the activation of the Notch pathway. Our results show that the A^465^T mutation is associated with an N1 LOF. This mutation was mostly found in squamous cell carcinoma (Table S2), where a prevalence of N1 LOF has been demonstrated (4).

The G^230^R (EGF6) mutation caused a strong decrease in *O-*fucosylation and *O-*fucose elongation but had very little effect on Notch signaling. The only visible effect was a loss of Notch inhibition induced by JAG1 in the presence of LFNG (Fig. 11). This result is consistent with previous studies where loss of *O-*fucosylation of EGF6 following mutation of the *O-*fucosylated threonine to valine showed similar results (23). This mutation would result in an N1 GOF in cases where JAG1 was the predominant activating ligand, and either LFNG or MFNG were expressed in the N1-expressing cells. Further studies are necessary to determine whether this is the case in the cancers listed in Table S2.

Although most of our signaling and binding assay results are explained by changes in glycosylation, some of them do not correlate or do not fully correlate with our mass spectral data. This is particularly the case for mutants G^309^R, D^464^N, and A^465^T. It cannot be excluded that the use of a truncated form of the N1 receptor (EGF5–14) with only five *O*-fucosylation sites presents a different *O*-glycosylation profile than the full-length form, which has 20 sites (17 of which are occupied) (23).

In conclusion, this study shows that mutations within the *O*-fucose consensus sequence of N1 EGFs can induce GOF or LOF being involved in the cancer process. It is very likely that similar mutations in other Notch receptors, especially NOTCH2, might have similar effects (33). It would also be interesting to study the effect of point mutations within the consensus sequences of other types of *O-*glycosylation (*e.g.*, *O*-glucose and *O*-GlcNAc) (18) of N1 found in some cancers. Overall, the essential role of *O-*glycosylation in the Notch pathway requires further investigation of their involvement in the cancer process.

## Experimental procedures

### Plasmid constructs

The plasmid encoding full-length human N1 (pcDNA3.1) was generously provided by Dr Steven Blacklow (Harvard) (49). Plasmids encoding human N1 EGF5–14 WT and mutants with C-terminal Myc-His_6_ tags (pSecTag2/HygroC; Invitrogen) was constructed by amplifying the ORF with primers: 5′-ATATATAAGCTTCACGGCAGGATGTCAACGAGTGTGG-3′ and 5′-ATCTAGCTCGAGCGATGTCCACCTCGCAGTGCGTC-3′. These primers include HindIII or XhoI restriction enzyme sites for subcloning, respectively, into pSecTag2/HygroC. All generated constructs were confirmed by DNA sequencing. The plasmid encoding LFNG (APtag2) was previously described (50). The TP1-1 luciferase reporter construct (Ga981-6) was a gift from Dr Georg Bornkamm (Munich, Germany), and the gWIZ-galactosidase construct was from Gene Therapy Systems. The red fluorescent protein (RFP) plasmid was obtained from Addgene (#12520).

### Mutagenesis

Full-length human N1 mutants were generated by using TaKaRa In-Fusion HD Cloning Plus system (Takara Bio, Inc). DNA fragments containing each mutated site were synthesized by Synbio Technologies (listed in Table S24) and amplified with CloneAmp HiFi PCR premix (Takara Bio, Inc). Amplification of each backbone fragment including vector portion was performed by using Platinum SuperFi II DNA polymerase (Invitrogen) with full-length human N1 pcDNA3 as a template. The primers used are listed in Table S25. All generated constructs were confirmed by DNA sequencing.

### Cell-based coculture N1 activation assay

Cell-based coculture N1 activation assay was performed in Pro5 CHO cells as previously described (24,51).

### Notch ligand–binding assays and cell surface N1 analysis

CHO cells were seeded in a 6-well tissue culture plate until reaching 70 to 80% confluence. Complete media were removed, cells were washed once with 1× PBS, and alpha-minimum essential medium without serum was added. CHO cells were transfected using 1 μg of pcDNA3 [N1] WT or mutants, 0.5 μg of pAPtag2 [EV] or [LFNG], and 0.3 μg of RFP plasmid using polyethyleneimine (with a polyethyleneimine/DNA ratio of 4/1). After 4 h, the media are removed, the cells are washed with 1× PBS, and complete media are added. After 24 h, cells are washed with 1× PBS and then 1 ml of 1× PBS is added. The cells are slowly detached by pipetting. After washing cells with washing buffer (Hank’s balanced salt solution), the cells were incubated with 100 nM DLL1-Fc (R&D Systems), JAG1-Fc (R&D Systems), or DLL4-Fc (R&D Systems) and anti-Fc phycoerythrin (PE)–conjugated antihuman antibody (Jackson ImmunoResearch; 1:20 dilution) or anti-Fc PE–conjugated antimouse antibody (Fisher Scientific; 1:20 dilution) in binding buffer on ice for 1 h (Hank’s balanced salt solution containing 1% bovine calf serum, 0.05% azide, and 1 mM CaCl_2_). For cell surface hN1 detection, washed cells were incubated with PE antihuman Notch 1 antibody (BioLegend; 1:20 dilution) on ice for 1 h. After washing cells with washing buffer, binding was determined and analyzed using an Accuri C6 flow cytometer. Ten thousand cells were gated for RFP expression, and PE intensity of the RFP-expressing cells was determined.

### Production of N1 EGF5–14 WT and mutants in the absence or the presence of LFNG

Production of N1 EGF5–14 WT was done in Pro5 CHO cells as previously described (24). Briefly, after approximately 5 days of production, media were collected centrifuged at 4000*g* for 15 min, and then syringe filtered with a 0.45 μm syringe filter. NaCl and imidazole were added to a final concentration of 1 M and 20 mM, respectively. For purification, a 150 μl nickel–nitrilotriacetic acid agarose (Qiagen) bead volume (300 μl 50% slurry) was used. Wash buffer consisted of 1 M NaCl and 20 mM imidazole in 1× Tris-buffered saline. Proteins were eluted using 250 mM imidazole in 1× Tris-buffered saline.

### Glycoproteomic mass spectral analysis of hN1 EGF5–14

Glycoproteomic analysis was performed as described previously (24). Briefly, 10 μl of purified protein was denatured and reduced using 10 μl of reducing buffer containing 8 M urea, 400 mM ammonium bicarbonate, and 10 mM Tris(2-carboxyethyl)phosphine (chymotrypsin digestion) or 8 M urea, 50 mM Tris–HCl, pH 8, and 10 mM Tris(2-carboxyethyl)phosphine (V8 digestion) at 50 °C for 5 min. Alkylation was performed at room temperature in the dark with 100 mM iodoacetamide in 50 mM Tris–HCl for 30 min. About 45 μl of mass spectral grade water (chymotrypsin digestion) or 250 mM diammonium phosphate solution (V8 digestion) were added to each sample. Chymotrypsin (50 ng) or V8 (50 ng) was added, and samples were incubated in a 37 °C water bath for 1 h (chymotrypsin) or 20 h (V8). Next, 7 μl of 5% formic acid were added, and samples were desalted with Millipore C18 Zip Tip Pipette Tips. After elution in 50% acetonitrile and 0.1% acetic acid, samples were diluted to 25% acetonitrile and and 0.1% formic acid. Approximately 10 ng of each sample were injected on a Q-Exactive Plus Orbitrap mass spectrometer (Thermo Fisher) with an Easy nano-LC HPLC system with a C18 EasySpray PepMap RSLC C18 column (50 μm × 15 cm; Thermo Fisher Scientific). A 30 min binary gradient solvent system (solvent A: 5% acetonitrile and 0.1% formic acid in water and solvent B: 80% acetonitrile and 0.1% formic acid in water) with a constant flow of 300 nl/min was used. Positive polarity mode was used with an *m/z* range of 400 to 2000 at a resolution of 70,000 and automatic gain control set to 1 × 10^6^. Higher energy collisional dissociation-tandem MS was used on the top 10 precursor ions in each full scan (collision energy set to 27%, 1 × 10^5^ gain control, isolation window *m/z* 1.2, dynamic exclusion enabled, and 17,500 fragment resolution). PMI-Byonic (version 2.10.5; Protein Metrics) was used to identify glycopeptides. Fixed modifications: carbamidomethyl +57.021464 at C. Variable modifications: oxidation +15.994915 at M, H, W, N, and D; deamidated +0.984016 at F, N, Q, and R. Glycoforms searched: unmodified peptide, modified peptide with *O*-fucose, modified peptide with *O*-fucose and HexNAc, modified peptide with *O*-fucose, HexNAc, and hexose, or modified peptide with *O*-fucose, HexNAc, hexose, and NeuAc. All these glycoforms were searched for in association with the presence or the absence of *O*-hexose, *O*-hexose and pentose, or *O*-hexose, pentose, and pentose. Precursor and fragment mass tolerance was set to 20 ppm. Four missed cleavages were allowed. The extracellular part of the human N1 receptor containing EGF5 to 14 (P46531) was used as a database. Xcalibur Qual Browser (version 2.0.3) was used to generate extracted ion chromatograms for all identified *O*-fucosylated peptides. For each peptide, the area under the curve was calculated for each peak corresponding to searched glycoforms. Relative abundance was calculated by comparing the area under the curve for a single glycoform to the total area under the curve for all searched glycoforms of a specific peptide. MS/MS spectra for each glycopeptide analyzed are shown in Fig. S6.

### Automated homology models for human N1 N^386^T, D^464^N, and A^465^T

Homology models were generated by using Swiss-model server for human N1 N^386^T, D^464^N, and A^465^T using structure of complex of N1 (EGF8–12) bound to Jagged1 (N-EGF3) (Protein Data Bank [PDB] code: 5UK5) or complex of N1 (EGF11–13) bound to Delta-like 4 (N-EGF2) (PDB code: 4XLW) as a reference template. Using Matchmaker of CHIMERA, these models were superimposed with the reference template (PDB codes: 5UK5 or 4XLW) to replace N1 WT by its mutated counterpart. Finally, a shortened version of the PDB file 5UK5 containing EGF12 with *O*-fucose was overlaid on EGF10 of the N^386^T mutant using Matchmaker of CHIMERA to allow the positioning of an *O*-fucose on this EGF.

### Statistical analysis

All experiments were performed in biological triplicates or more, and results were reported as the means ± SD. Statistical significance was determined using unpaired *t* test. Significance levels: (∗∗∗) for *p* < 0.005, (∗∗) for *p* < 0.001, and (∗) for *p* < 0.05.

## Data availability

The MS proteomics data have been deposited to the ProteomeXchange Consortium *via* the PRIDE (52) partner repository (https://www.ebi.ac.uk/pride/accessed on August 26, 2022) with the dataset identifier PXD036345. Table S23 provides a description of the files in the PRIDE repository.

## Supporting information

This article contains supporting information.

## Conflict of interest

The authors declare that they have no conflicts of interest with the contents of this article.
