# Supplementary material for: Cancer-associated Notch receptor variants lead to O-fucosylation defects that deregulate Notch signaling
Source: J Biol Chem. 2022 Oct 18;298(12):102616. doi: 10.1016/j.jbc.2022.102616 (PMC9672452; doi:10.1016/j.jbc.2022.102616)
Supplement: Supporting Information [file mmc1.pdf]

Supporting Information for:

Cancer-associated Notch receptor variants lead to *O*-fucosylation defects that deregulate Notch signaling

Florian Pennarubia, Atsuko Ito, Megumi Takeuchi, Robert S Haltiwanger

Contents:

Tables S1-S25

Figures S1-S6

EGF	Mutation	Frequency Biomuta	Frequency COSMIC	EGF	Mutation	Frequency Biomuta	Frequency COSMIC
6	C^{227}F		1	9	A^{348}D		1
	G^{230}E		1		A^{348}V		2
	G^{230}R		3		A^{348}T		1
	G^{231}S		1		T^{349}P	1	35
	T^{232}I		1		T^{349}I	2	
	R^{234}C	1	1		R^{353}C	7	9
	C^{243}Y		1		R^{365}C	3	12
	C^{243}R		1	10	N^{386}T		1
	C^{254}R		1	12	C^{456}Y	6	12
8	C^{306}Y		5		C^{461}Y	3	2
	G^{309}R	1			C^{461}W		1
	G^{310}R	8	11		N^{463}S		1
	G^{310}E	1			D^{464}N		1
	T^{311}P	1	42		A^{465}T	9	18
	T^{311}N		1		T^{466}I		1
	G^{317}C	1			T^{466}A		1
	C^{323}S	1	1		C^{467}W	1	
	C^{323}F		1		C^{467}Y	4	3
	W^{327}C	1	6		C^{467}F	5	1
	C^{332}Y	1	2		C^{467}G		3
9	C^{339}S	2			C^{478}Y	4	2
	C^{339}F		1		C^{478}S	2	1
	C^{344}F	2	1		C^{478}F		4
	G^{347}S	3	4		G^{481}C	3	1
	G^{347}C		1		C^{487}Y		1

Table S1. List and frequency of N1 receptor mutations that can affect the *O*-fucosylation of EGF6 to 13. Single point mutations within the *O*-fucose consensus sequence in EGF6-13 of the N1 receptor were manually searched in the COSMIC and Biomuta databases.

EGF	Mutation	Tissue type	Cancer type	Frequency	Zygosity
6	$G^{230}R$	Haematopoietic and lymphoid	Acute leukaemic transformation of myeloproliferative neoplasm	1	Unknown
			Myeloproliferative neoplasm	1	Unknown
8	$G^{309}R$	Stomach		1	Unknown
	$G^{310}R$	Brain	Glioma	1	Unknown
			Oligodendroglioma Grade III	1	Heterozygous
		Oesophagus	Squamous cell carcinoma	2	Unknown
		Penis	Squamous cell carcinoma	1	Unknown
		Skin	Malignant melanoma	1	Unknown
			Squamous cell carcinoma	2	Heterozygous
		Head neck	Squamous cell carcinoma	1	Unknown
				1	Heterozygous
		Mouth		1	Unknown
	$T^{311}P$	Haematopoietic and lymphoid	Acute lymphoblastic T cell leukaemia	1	Unknown
			Chronic myelomonocytic leukaemia	1	Unknown
		Head neck	Squamous cell carcinoma	23	Unknown
		Large intestine	Adenocarcinoma	1	Unknown
		Thyroid	Carcinoma	8	Unknown
			Neoplasm	1	Unknown
		Striated muscle	Embryonal	2	Unknown
			Alveolar	5	Unknown
9	$G^{347}S$	Colon	Adenocarcinoma	1	Unknown
		Lung	Adenocarcinoma	1	Unknown
				1	Heterozygous
		Head neck	Squamous cell carcinoma	1	Unknown
	$T^{349}P$	Central nervous system	Primary central nervous system lymphoma	2	Unknown
		Haematopoietic and lymphoid	Acute lymphoblastic T cell leukaemia	3	Unknown
			Chronic lymphocytic leukaemia-small lymphocytic lymphoma	1	Unknown
			Chronic myelomonocytic leukaemia	1	Unknown
		Thyroid	Carcinoma	4	Unknown
			Neoplasm	4	Unknown
		Head neck	Squamous cell carcinoma	20	Unknown
10	$N^{386}T$	Haematopoietic and lymphoid	Acute lymphoblastic T cell leukaemia	1	Unknown
12	$D^{464}N$	Mouth	Carcinoma	1	Unknown
		Breast		1	Unknown
	$A^{465}T$	Brain	Oligodendroglioma Grade III	1	Heterozygous
				2	Unknown
			Glioma	1	Unknown
		Endometrium	Endometrioid carcinoma	1	Unknown
		Colon	Adenocarcinoma	1	Unknown
		Large intestine	Adenocarcinoma	1	Unknown
		Oesophagus	Squamous cell carcinoma	2	Unknown
			Squamous cell carcinoma	1	Unknown
			Squamous cell carcinoma	1	Unknown
			Carcinoma	1	Unknown
			Adenocarcinoma	1	Unknown
		Skin	Basal cell carcinoma	1	Unknown
			Squamous cell carcinoma	1	Heterozygous
			Squamous cell carcinoma	1	Unknown
		Head neck	Squamous cell carcinoma	1	Unknown
		Mouth	Squamous cell carcinoma	2	Unknown

Table S2. Origin, frequency, and zygosity of selected mutations. For each of the nine selected mutations, the tissue and cancer type where the mutation has been detected, frequency, and zygosity were manually searched in the COSMIC and Biomuta databases.

Table S3: Chymotrypsin or V8 digestion of hN1 EGF5-14 WT + EV analyzed for Figure 2 to 10, S1 and, S3.

Table S4: Chymotrypsin or V8 digestion of hN1 EGF5-14 WT + LFNG analyzed for Figure 2 to 10, S1 and, S3.

Table S5: V8 digestion of hN1 EGF5-14 $G^{230}R$ + EV analyzed for Figure 2.

Table S6: V8 digestion of hN1 EGF5-14 $G^{230}R$ + LFNG analyzed for Figure 2.

Table S7: Chymotrypsin digestion of hN1 EGF5-14 $G^{309}R$ + EV analyzed for Figure 3.

Table S8: Chymotrypsin digestion of hN1 EGF5-14 $G^{309}R$ + LFNG analyzed for Figure 3.

Table S9: Chymotrypsin digestion of hN1 EGF5-14 $G^{310}R$ + EV analyzed for Figure 4.

Table S10: Chymotrypsin digestion of hN1 EGF5-14 $G^{310}R$ + LFNG analyzed for Figure 4.

Table S11: Chymotrypsin digestion of hN1 EGF5-14 $T^{311}P$ + EV analyzed for Figure 5.

Table S12: Chymotrypsin digestion of hN1 EGF5-14 $T^{311}P$ + LFNG analyzed for Figure 5.

Table S13: Chymotrypsin digestion of hN1 EGF5-14 $G^{347}S$ + EV analyzed for Figure 6.

Table S14: Chymotrypsin digestion of hN1 EGF5-14 $G^{347}S$ + LFNG analyzed for Figure 6.

Table S15: Chymotrypsin digestion of hN1 EGF5-14 $T^{349}P$ + EV analyzed for Figure 7.

Table S16: Chymotrypsin digestion of hN1 EGF5-14 $T^{349}P$ + LFNG analyzed for Figure 7.

Table S17: Chymotrypsin or V8 digestion of hN1 EGF5-14 $N^{386}T$ + EV analyzed for Figure 8 and S1.

Table S18: Chymotrypsin or V8 digestion of hN1 EGF5-14 $N^{386}T$ + LFNG analyzed for Figure 8 and S1.

Table S19: Chymotrypsin or V8 digestion of hN1 EGF5-14 $D^{464}N$ + EV analyzed for Figure 9 and S3.

Table S20: Chymotrypsin or V8 digestion of hN1 EGF5-14 $D^{464}N$ + LFNG analyzed for Figure 9 and S3.

Table S21: V8 digestion of hN1 EGF5-14 $A^{465}T$ + EV analyzed for Figure 10.

Table S22: V8 digestion of hN1 EGF5-14 $A^{465}T$ + LFNG analyzed for Figure 10.

Table S23: Description of the files uploaded to the PRIDE repository.

Table S24: List of synthesized DNA fragment for hNotch1 mutants construct

Table S25: List of Primers used for hNotch1 mutants construct

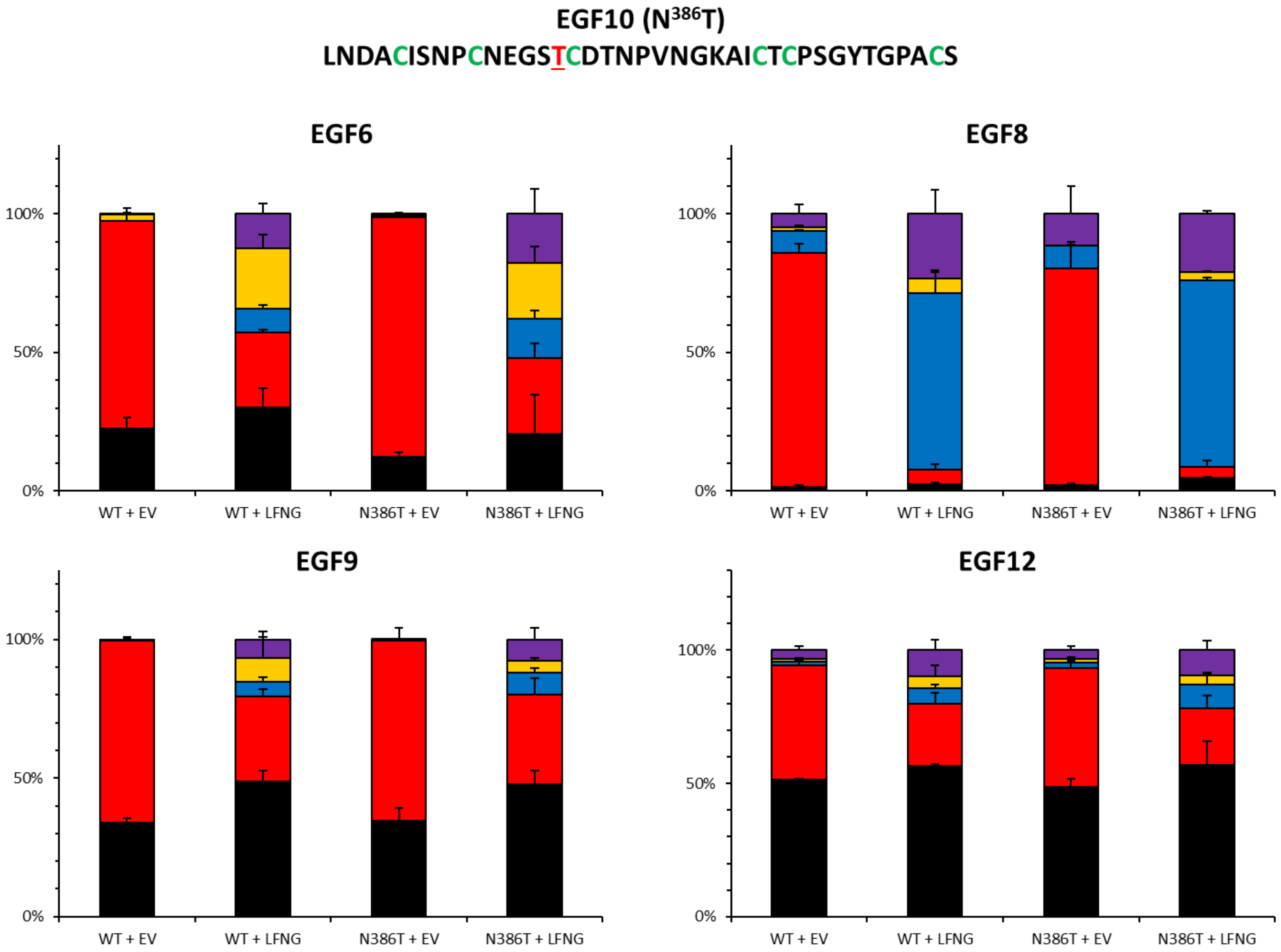

Figure S1. $N^{386}T$ mutant in EGF10 had no effect on *O*-fucosylation of other EGFs. Quantification of the percentage of unmodified and *O*-fucosylated peptides for the EGF6, 8, 9 and 12 as described in Experimental Procedures (n=2 or 3). Unmodified peptide (black), modified by a monosaccharide *O*-fucose (red), *O*-fucose + GlcNAc (blue), *O*-fucose + GlcNAc + galactose (yellow) and *O*-fucose + GlcNAc + galactose + sialic acid (purple). The data used to generate the EICs are available in the Table S3, S4, S17 and S18.

EGF10 (N^{386}T)

LNDACISNPCNEGSTCDTNPVNGKAICTCPSGYTGPACS

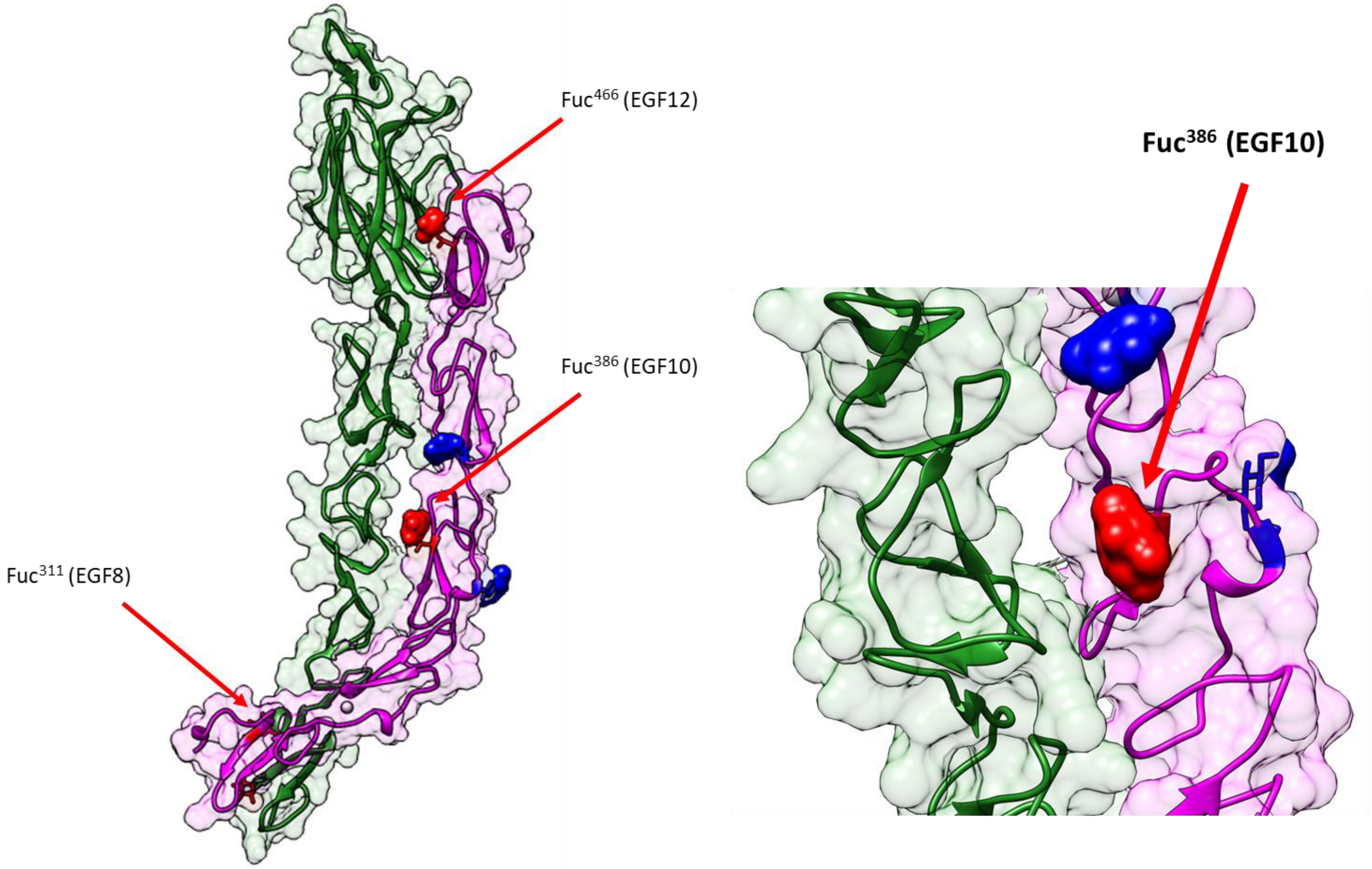

Figure S2. Automated homology model for human N1 N^{386}T suggests neo O-fucose on EGF10 could enhance interaction with JAG1. Homology model was generated as described in Experimental Procedures. hN1 N^{386}T (purple), JAG1 (green), *O*-glucose (blue), *O*-fucose (red).

EGF12 ($D^{464}N$)

DVNECVSNPCQNNATCLDQIGEFQCICMPGYEGVHCE

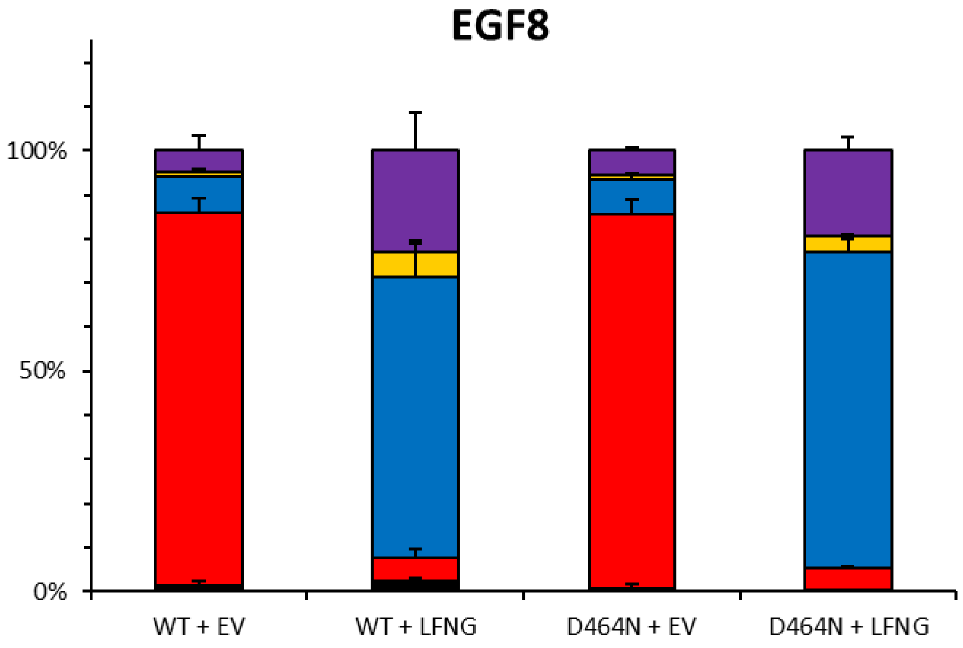

Figure S3. $D^{464}N$ mutation in EGF12 had no effect on EGF8 *O*-fucosylation. Quantification of the percentage of unmodified and *O*-fucosylated peptides for the EGF8 as described in Experimental Procedures (n = 2). Unmodified peptide (black), modified by a monosaccharide *O*-fucose (red), *O*-fucose + GlcNAc (blue), *O*-fucose + GlcNAc + galactose (yellow) and *O*-fucose + GlcNAc + galactose + sialic acid (purple). The data used to generate the EICs are available in the Table S3, S4, S19, S20.

EGF12 (D^{464}N)

DVNECVSNPCQNNATCLDQIGEFQCICMPGYEGVHCE

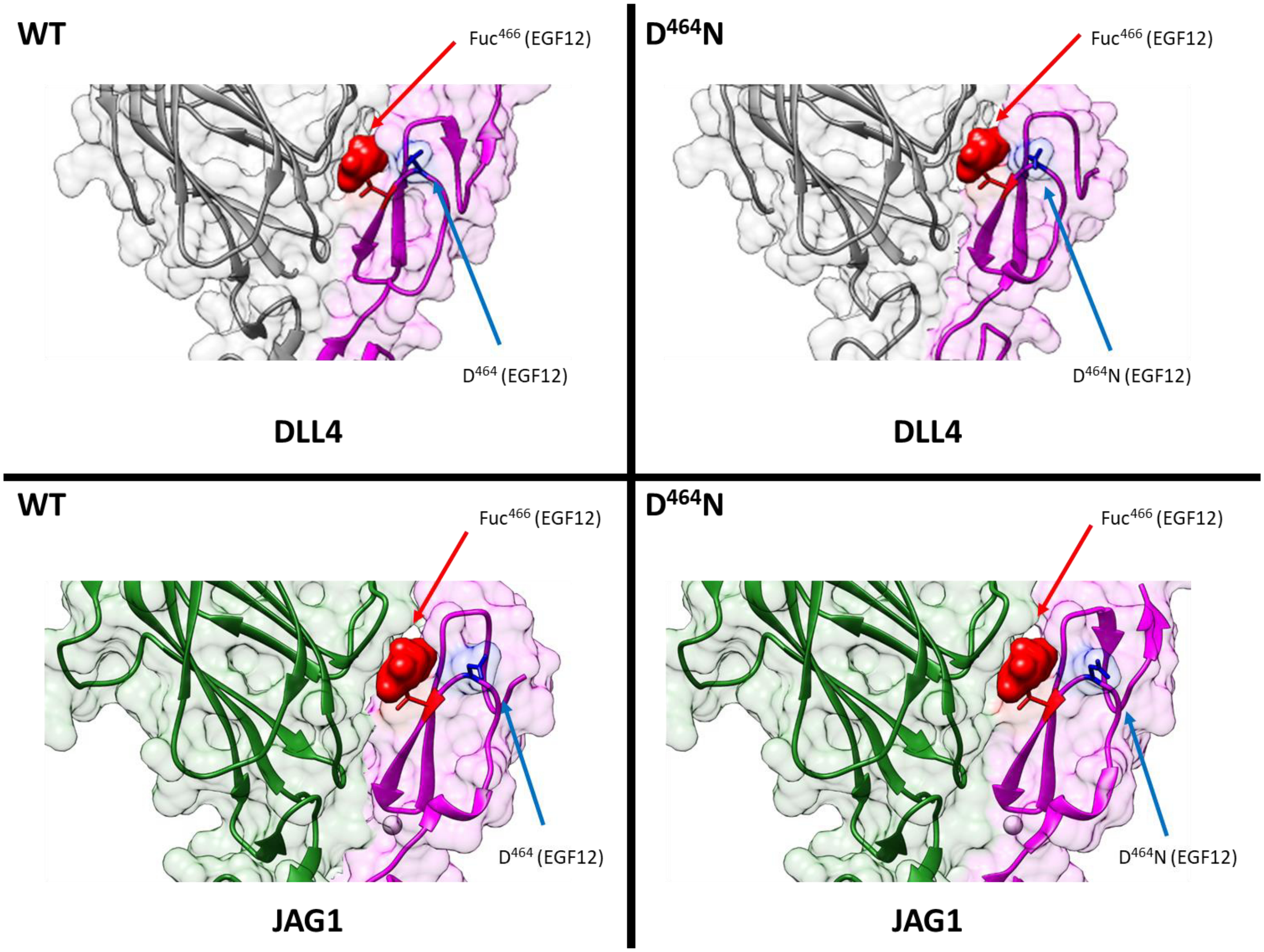

Figure S4. Automated homology models for human N1 D^{464}N suggest that the asparagine in not in direct contact with DLL4 or JAG1. Homology model was generated as described in Experimental Procedures. hN1 (purple), DLL4 (grey), JAG1 (green), *O*-fucose (red), mutation site at position 464 (blue).

EGF12 ($D^{464}N$)

DVNECVSNPCQNDTTCLDQIGEFQCICMPGYEGVHCE

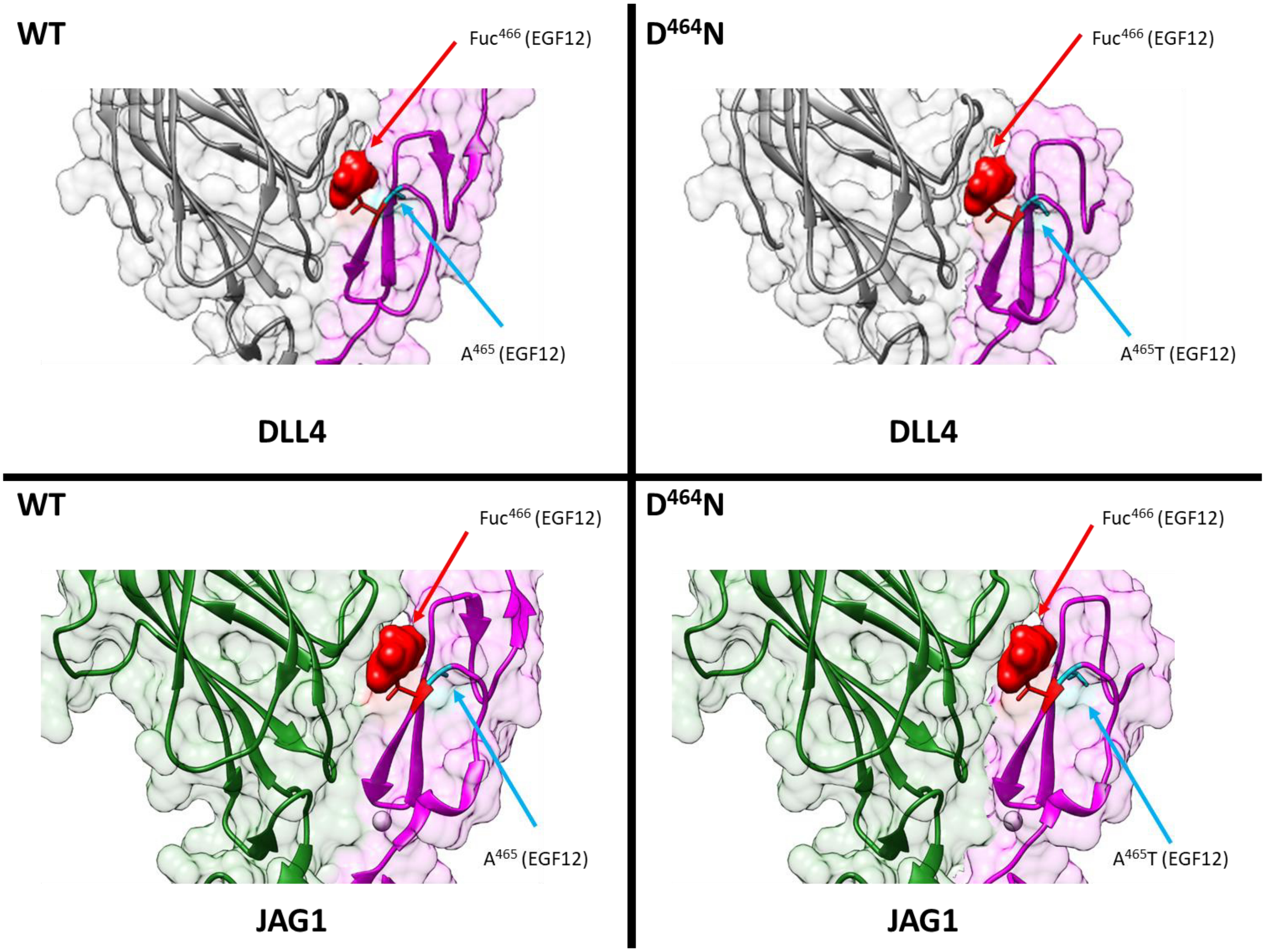

Figure S5. Automated homology models for human N1 $A^{465}T$ suggest that the threonine is not in direct contact with DLL4 or JAG1. Homology model was generated as described in Experimental Procedures. hN1 (purple), DLL4 (grey), JAG1 (green), *O*-fucose (red), mutation site at position 465 (blue).

Figure S6A

EGF 6 : [22]VGSYRCVCRATHTGPNCERPYVPCSPSPCQNGGTCRPTGDVTHE[65]

#	a calc.	a obs.	b calc.	b obs.	b-18 calc.	b-18 obs.	b++ calc.	b++ obs.	b_3+ calc.	b_3+ obs.	Seq.	y calc.	y obs.	y++ calc.	y++ obs.	y_3+ calc.	y_3+ obs.	#
1	72.0808		100.0757		82.0651		50.5415		34.0301		V							44
2	129.1022		157.0971		139.0866		79.0522		53.0372		G	5447.282		2724.145		1816.432		43
3	216.1343		244.1292		226.1186		122.5682		82.0479		S	5390.261		2695.634		1797.425		42
4	379.1976		407.1925		389.1819		204.0999		136.4024		Y	5303.229		2652.118		1768.414		41
5	535.2987		563.2936		545.2831		282.1504		188.4361		R	5140.165		2570.586		1714.06		40
6	695.3293		723.3243		705.3137		362.1658		241.7796		C	4984.064		2492.536		1662.026		39
7	794.3978		822.3927		804.3821		411.7		274.8024		V	4824.033		2412.52		1608.683		38
8	954.4284		982.4233		964.4127		491.7153		328.146		C	4724.965		2362.986		1575.66		37
9	1110.53		1138.524		1120.514		569.7658		380.1797		R	4564.934		2282.971		1522.316		36
10	1181.567		1209.562		1191.551		605.2844		403.8587		A	4408.833		2204.92		1470.283		35
11	1282.614		1310.609		1292.599		655.8082		437.5413		T	4337.796		2169.402		1446.604		34
12	1419.673		1447.668		1429.658		724.3377	724.3472	483.2276		H	4236.749		2118.878		1412.921		33
13	1520.721		1548.716		1530.705		774.8615		516.9101		T	4099.69		2050.348		1367.235		32
14	1577.742		1605.737		1587.727		803.3723		535.9173		G	3998.642		1999.825		1333.552		31
15	1674.795		1702.79		1684.78		851.8986		568.2682		P	3941.62		1971.314		1314.545		30
16	1788.838		1816.833		1798.822		908.9201		606.2825		N	3844.568		1922.788		1282.194		29
17	1948.869		1976.864		1958.853		988.9354		659.626		C	3730.525		1865.766		1244.18		28
18	2077.911		2105.906		2087.896		1053.457		702.6402		E	3570.494		1785.751		1190.836		27
19	2234.012		2262.007		2243.997		1131.507		754.6739		R	3441.452		1721.229		1147.822		26
20	2331.065		2359.06		2341.05		1180.034		787.0249		P	3285.35		1643.179		1095.788		25
21	2494.129		2522.123		2504.113		1261.565		841.3793		Y	3188.298		1594.652		1063.437		24
22	2593.197		2621.192		2603.181		1311.1		874.4021	874.3983	V	3025.234		1513.121		1009.083		23
23	2690.25		2718.245		2700.234		1359.626		906.753		P	2926.166		1463.587		976.0601		22
24	2850.28		2878.275		2860.265		1439.641		960.0966		C	2829.113		1415.06		943.7092		21
25	3448.502		3476.497		3458.487		1738.752		1159.504		S	2669.083		1335.045		890.3657		20
26	3545.555		3573.55		3555.54		1787.279		1191.855		P	2070.86		1035.934		690.9583		19
27	3632.587		3660.582		3642.572		1830.795		1220.866		S	1973.808		987.4074		658.6074		18
28	3729.64		3757.635		3739.624		1879.321		1253.217		P	1886.776	1886.801	943.8914	943.8904	629.5967	629.5969	17
29	3889.671		3917.666		3899.655		1959.336		1306.56		C	1789.723		895.365		597.2458	597.2346	16
30	4017.729		4045.724		4027.714		2023.366		1349.246		Q	1629.692		815.3497	815.347	543.9022		15
31	4132.756		4160.751		4142.741		2080.879		1387.589	1387.597	N	1501.634	1501.636	751.3204	751.3171	501.216		14
32	4189.778		4217.773		4199.762		2109.39		1406.596		G	1386.607	1386.611	693.8069	693.8043	462.8737		13
33	4246.799		4274.794		4256.783		2137.901		1425.603		G	1329.585		665.2962		443.8666		12
34	4347.847		4375.842		4357.831		2188.425		1459.285		T	1272.564		636.7855		424.8594		11
35	4507.877		4535.872		4517.862		2268.44		1512.629		C	1171.516		586.2616		391.1769		10
36	4663.979		4691.973		4673.963		2346.49		1564.663		R	1011.485		506.2463		337.8333		9
37	4761.031		4789.026		4771.016		2395.017		1597.014		P	855.3843		428.1958		285.7996		8
38	4862.079		4890.074		4872.063		2445.541		1630.696		T	758.3315		379.6694		253.4487		7
39	4919.1		4947.095		4929.085		2474.051		1649.703		G	657.2838		329.1456		219.7661		6
40	5034.127		5062.122		5044.112		2531.565		1688.046		D	600.2624		300.6348		200.759		5
41	5133.196		5161.191		5143.18		2581.099		1721.068		V	485.2354	485.234	243.1214		162.4167		4
42	5234.243		5262.238		5244.228		2631.623		1754.751		T	386.167	386.1635	193.5871		129.3939		3
43	5371.302		5399.297		5381.287		2700.152		1800.437		H	285.1193	285.118	143.0633		95.7113		2
44											E	148.0604		74.5339		50.025		1

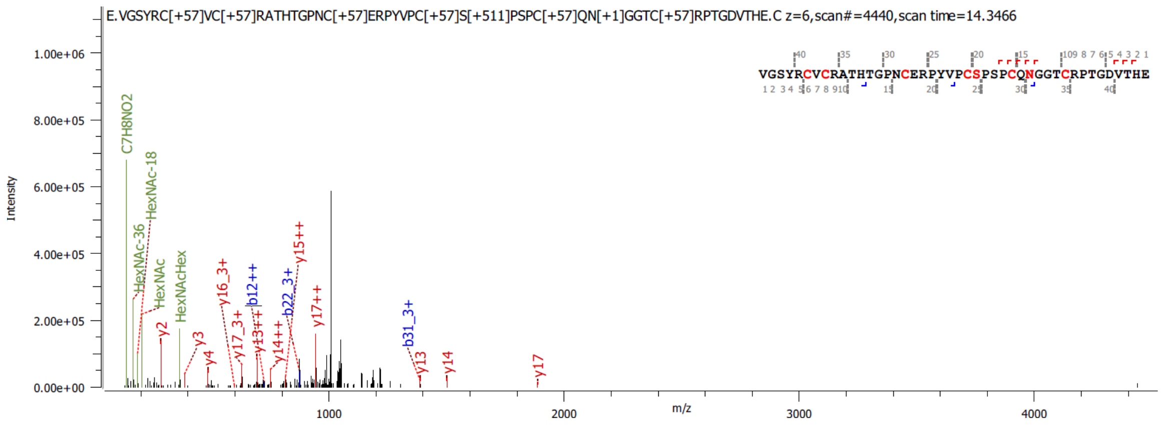

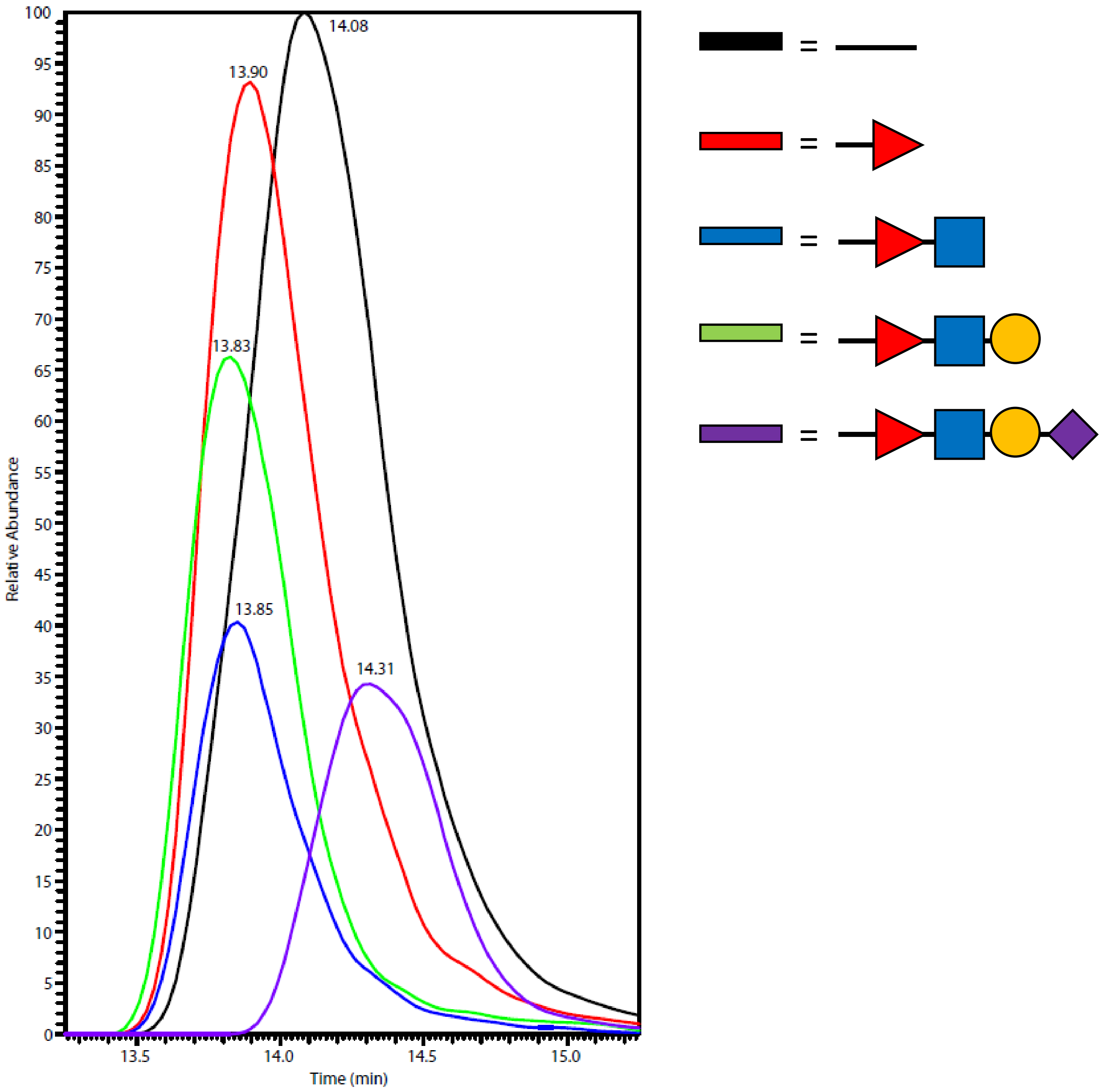

Figure S6B

EGF 8 : 103NCRCPPEWTGQYCTEDVDECQLMPNACQNGGTCHNTHGGY142

#	a calc.	a obs.	b calc.	b obs.	b-18 calc.	b-18 obs.	b++ calc.	b++ obs.	Seq.	y calc.	y obs.	y++ calc.	y++ obs.	y_3+ calc.	y_3+ obs.	#
1	87.0553		115.0502		97.0396		58.0287		N							40
2	247.0859		275.0808		257.0703		138.0441		C	5478.08		2739.543		1826.698		39
3	403.187		431.182	431.1824	413.1714		216.0946		R	5318.049		2659.528		1773.355		38
4	563.2177	563.215	591.2126	591.2145	573.202		296.1099		C	5161.948		2581.478		1721.321		37
5	660.2704		688.2654		670.2548		344.6363		P	5001.917		2501.462		1667.977		36
6	757.3232		785.3181	785.3146	767.3076		393.1627		P	4904.864		2452.936		1635.626		35
7	886.3658		914.3607	914.3623	896.3501		457.684		E	4807.812		2404.41		1603.275		34
8	1072.445		1100.44	1100.444	1082.43		550.7236		W	4678.769		2339.888		1560.261		33
9	1173.493		1201.488	1201.491	1183.477		601.2475		T	4492.69		2246.849		1498.235		32
10	1230.514		1258.509		1240.499		629.7582		G	4391.642		2196.325		1464.552		31
11	1358.573		1386.568	1386.575	1368.557		693.7875	693.7838	Q	4334.621		2167.814		1445.545		30
12	1521.636		1549.631		1531.621		775.3192		Y	4206.562		2103.785		1402.859		29
13	1681.667		1709.662		1691.651		855.3345	855.3192	C	4043.499		2022.253		1348.504		28
14	1782.715		1810.709		1792.699		905.8583		T	3883.468		1942.238		1295.161		27
15	1911.757		1939.752	1939.715	1921.741		970.3796		E	3782.42		1891.714		1261.478		26
16	2026.784		2054.779	2054.808	2036.768		1027.893		D	3653.378		1827.193		1218.464		25
17	2125.852		2153.847	2153.861	2135.837		1077.427		V	3538.351		1769.679		1180.122		24
18	2240.879		2268.874	2268.882	2250.864		1134.941		D	3439.282		1720.145		1147.099		23
19	2369.922		2397.917	2397.898	2379.906		1199.462		E	3324.256		1662.631		1108.757		22
20	2529.953		2557.948		2539.937		1279.477		C	3195.213		1598.11		1065.743		21
21	2658.011		2686.006	2686.007	2667.996		1343.507		Q	3035.182		1518.095		1012.399		20
22	2771.095		2799.09		2781.08		1400.049		L	2907.124		1454.066		969.7127		19
23	2902.136		2930.131		2912.12		1465.569	1465.581	M	2794.04		1397.524		932.0181		18
24	2999.189		3027.183		3009.173		1514.095		P	2662.999		1332.003		888.3379		17
25	3113.231		3141.226		3123.216		1571.117		N	2565.946		1283.477		855.987		16
26	3184.269		3212.263		3194.253		1606.635		A	2451.904		1226.455		817.9727		15
27	3344.299		3372.294		3354.284		1686.651		C	2380.866		1190.937		794.2936		14
28	3472.358		3500.353		3482.342		1750.68		Q	2220.836		1110.922		740.9501		13
29	3586.401		3614.396		3596.385		1807.701		N	2092.777		1046.892		698.2639		12
30	3643.422		3671.417		3653.407		1836.212		G	1978.734		989.8707		660.2496		11
31	3700.444		3728.439		3710.428		1864.723		G	1921.713		961.36		641.2424		10
32	4603.777		4631.772		4613.761		2316.39		T	1864.691		932.8493		622.2353		9
33	4763.807		4791.802		4773.792		2396.405		C	961.3581		481.1827		321.1242		8
34	4900.866		4928.861		4910.851		2464.934		H	801.3274	801.3216	401.1674		267.7807		7
35	5030.904		5058.899		5040.889		2529.953		N	664.2685	664.2712	332.6379		222.0944		6
36	5131.952		5159.947		5141.936		2580.477		T	534.2307	534.2313	267.619		178.7484		5
37	5269.011		5297.006		5278.995		2649.007		H	433.183	433.1853	217.0951		145.0659		4
38	5326.032		5354.027		5336.017		2677.517		G	296.1241		148.5657		99.3795		3
39	5383.054		5411.049		5393.038		2706.028		G	239.1026		120.055		80.3724		2
40									Y	182.0812		91.5442		61.3652		1

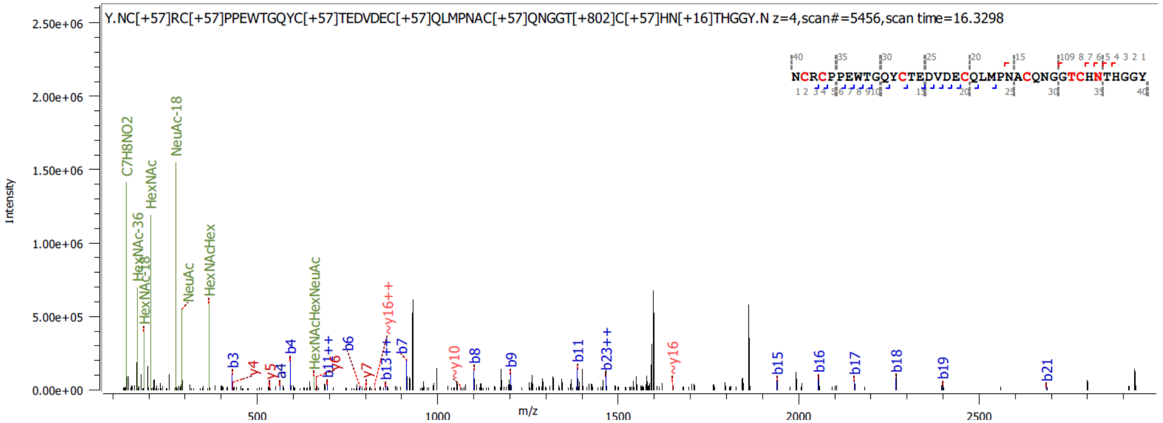

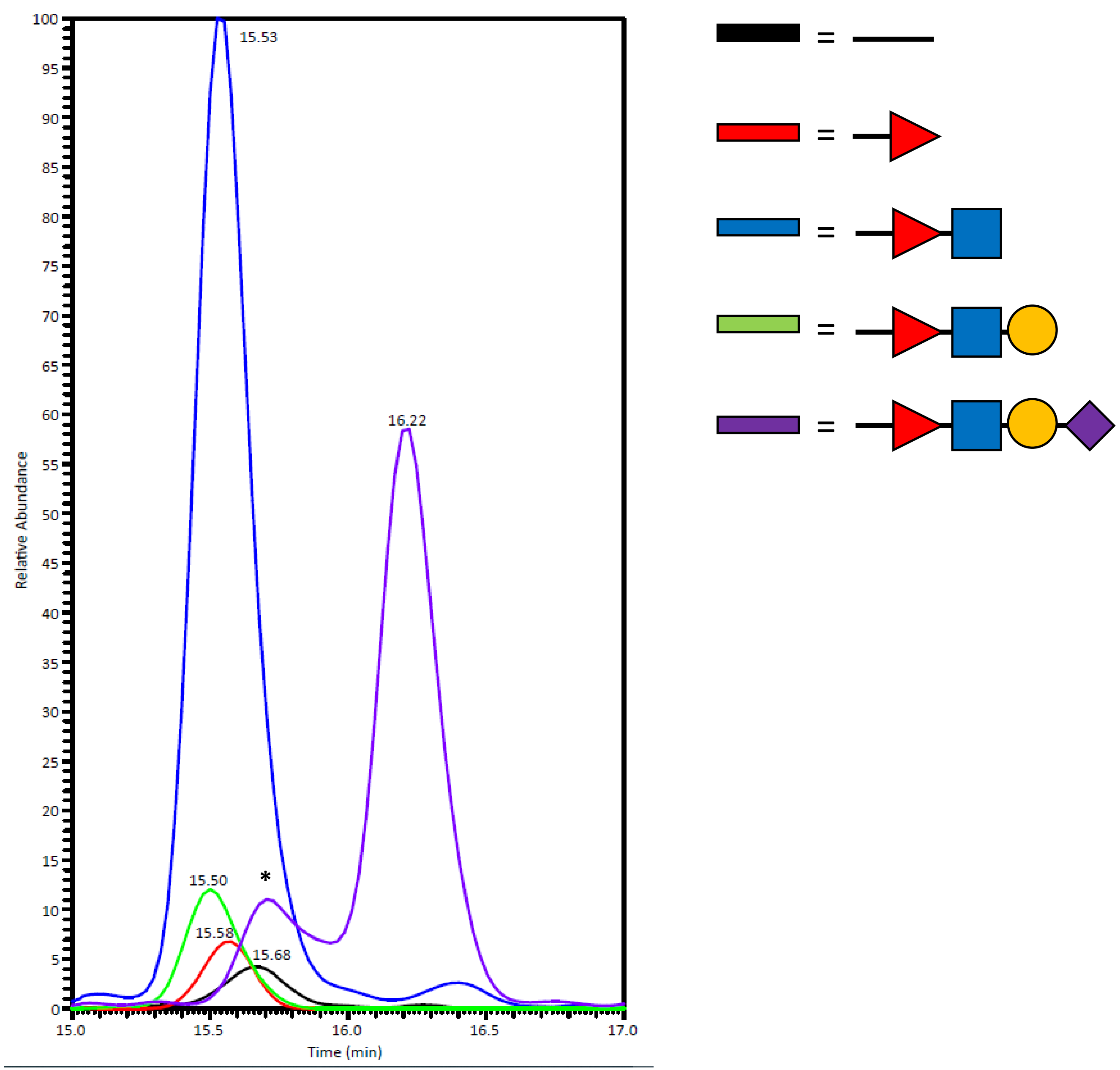

Figure S6C

EGF 9 : 169HGATCHDRVASF180

#	a calc.	a obs.	b calc.	b obs.	b-18 calc.	b-18 obs.	b++ calc.	b++ obs.	Seq.	y calc.	y obs.	y++ calc.	y++ obs.	#
1	110.0713		138.0662		120.0556		69.5367		H					12
2	167.0927	167.0923	195.0876	195.0883	177.0771		98.0475		G	1366.606		683.8064		11
3	238.1298		266.1248	266.1254	248.1142		133.566		A	1309.584		655.2957		10
4	339.1775		367.1724	367.1736	349.1619		184.0899		T	1238.547		619.7771		9
5	499.2082		527.2031		509.1925		264.1052		C	1137.499		569.2533		8
6	636.2671		664.262		646.2514		332.6346		H	977.4687		489.238		7
7	751.294		779.2889	779.2925	761.2784	761.2827	390.1481		D	840.4098		420.7085		6
8	907.3951		935.39		917.3795		468.1987	468.2002	R	725.3828		363.195		5
9	1006.464		1034.458		1016.448		517.7329		V	569.2817		285.1445		4
10	1077.501		1105.496	1105.494	1087.485		553.2514	553.2532	A	470.2133		235.6103		3
11	1310.591		1338.586		1320.575		669.7964		S	399.1762		200.0917		2
12									F	166.0863	166.0868	83.5468		1

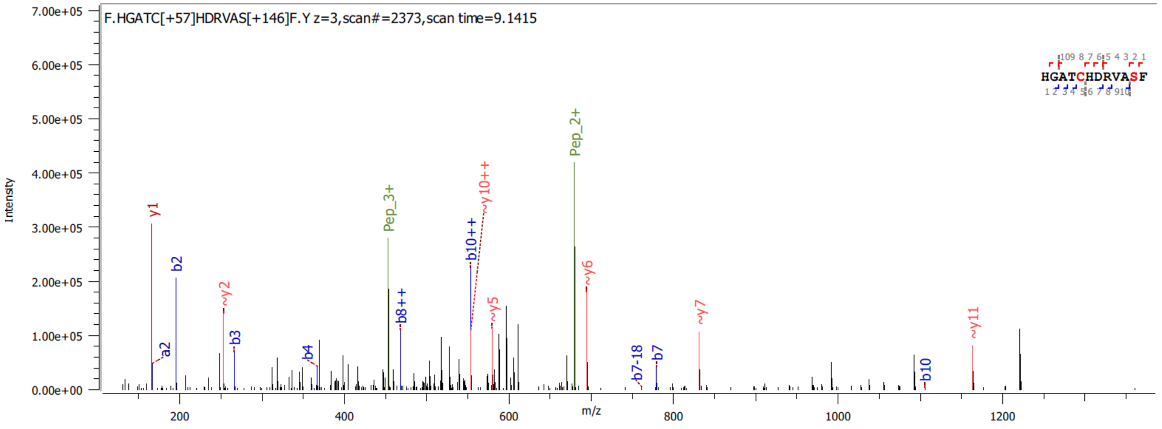

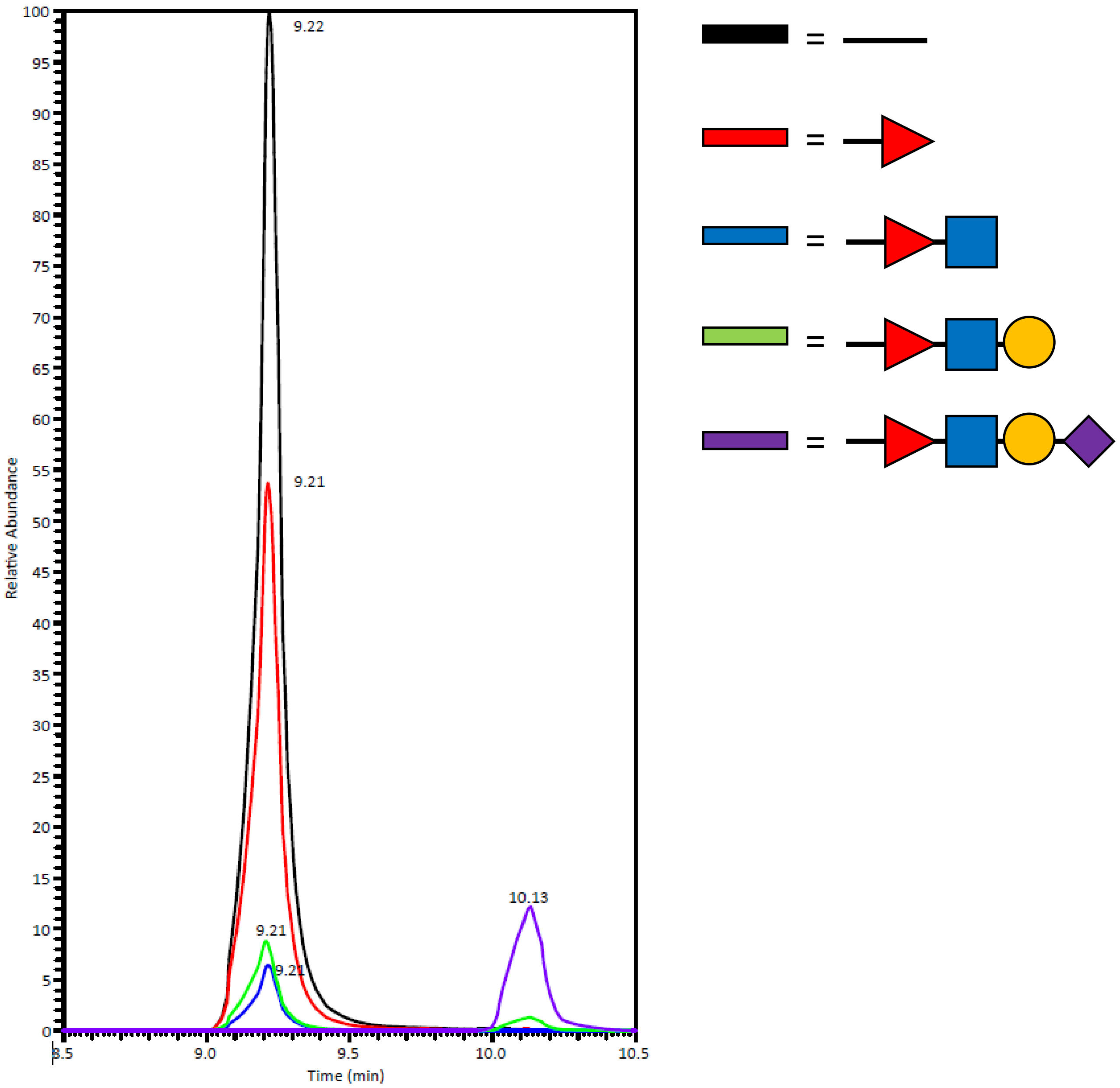

Figure S6D

EGF 10 : ^{207}GSNCDTNPVNGKAICTCPSGYTGPACSQDVDE^{238}

#	a calc.	a obs.	b calc.	b obs.	b-18 calc.	b-18 obs.	b++ calc.	b++ obs.	Seq.	y calc.	y obs.	y++ calc.	y++ obs.	#
1	30.0338		58.0287		40.0182		29.518		G					32
2	117.0659		145.0608		127.0502		73.034		S	3375.35		1688.179		31
3	231.1088		259.1037		241.0931		130.0555		N	3288.318		1644.663		30
4	391.1394		419.1343		401.1238		210.0708		C	3174.275		1587.641		29
5	506.1664		534.1613	534.159	516.1507		267.5843		D	3014.245		1507.626		28
6	607.214		635.209	635.2066	617.1984		318.1081		T	2899.218		1450.113		27
7	721.257		749.2519	749.246	731.2413	731.227	375.1296		N	2798.17		1399.589		26
8	818.3097		846.3046		828.2941		423.656		P	2684.127		1342.567		25
9	917.3781		945.3731	945.3707	927.3625	927.3552	473.1902		V	2587.075		1294.041		24
10	1032.405		1060.4	1060.404	1042.389		530.7036		N	2488.006		1244.507		23
11	1089.427		1117.422		1099.411		559.2144		G	2372.979		1186.993		22
12	1217.522		1245.516		1227.506		623.2619		K	2315.958		1158.483		21
13	1288.559		1316.554	1316.554	1298.543	1298.552	658.7804		A	2187.863		1094.435		20
14	1401.643		1429.638	1429.637	1411.627	1411.619	715.3224		I	2116.826		1058.916		19
15	1561.673		1589.668	1589.68	1571.658		795.3378		C	2003.742		1002.374		18
16	1662.721		1690.716	1690.708	1672.705		845.8616		T	1843.711		922.3591		17
17	1822.752		1850.747	1850.745	1832.736	1832.772	925.8769		C	1742.663	1742.656	871.8352		16
18	1919.804		1947.799		1929.789		974.4033		P	1582.633	1582.619	791.8199		15
19	2006.836		2034.831		2016.821		1017.919		S	1485.58		743.2935		14
20	2063.858		2091.853		2073.842		1046.43		G	1398.548	1398.539	699.7775		13
21	2226.921		2254.916		2236.906		1127.962		Y	1341.526		671.2668		12
22	2327.969		2355.964		2337.953		1178.486		T	1178.463	1178.463	589.7351		11
23	2384.99		2412.985		2394.975		1206.996		G	1077.415	1077.413	539.2113		10
24	2482.043		2510.038		2492.028		1255.523		P	1020.394	1020.387	510.7006		9
25	2553.08		2581.075		2563.065		1291.041		A	923.3411		462.1742		8
26	2713.111		2741.106		2723.095		1371.057		C	852.304	852.3055	426.6556		7
27	2800.143		2828.138		2810.127		1414.573		S	692.2733	692.2692	346.6403		6
28	2928.202		2956.196		2938.186		1478.602		Q	605.2413		303.1243		5
29	3043.228		3071.223		3053.213		1536.115		D	477.1827		239.095		4
30	3142.297		3170.292		3152.281		1585.65		V	362.1558	362.1518	181.5815		3
31	3257.324		3285.319		3267.308		1643.163		D	263.0874	263.0861	132.0473		2
32									E	148.0604	148.0599	74.5339		1

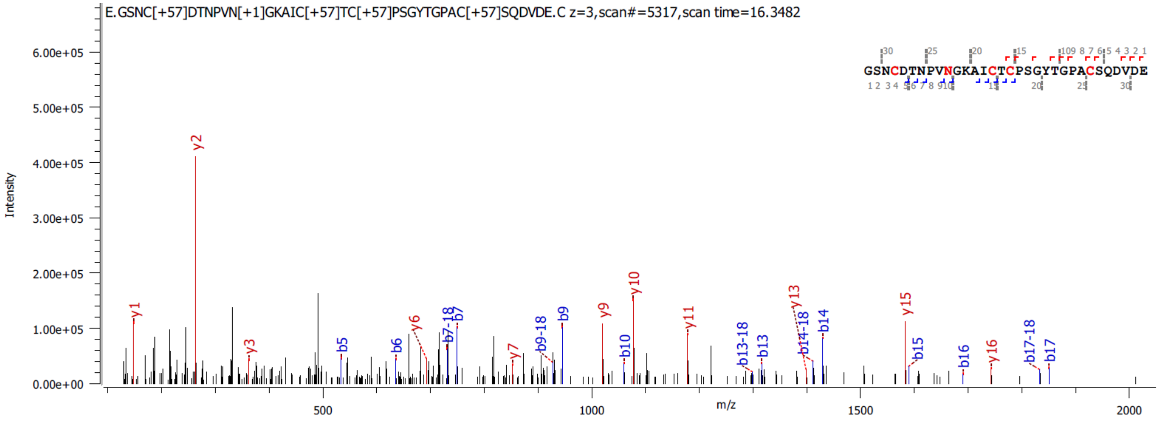

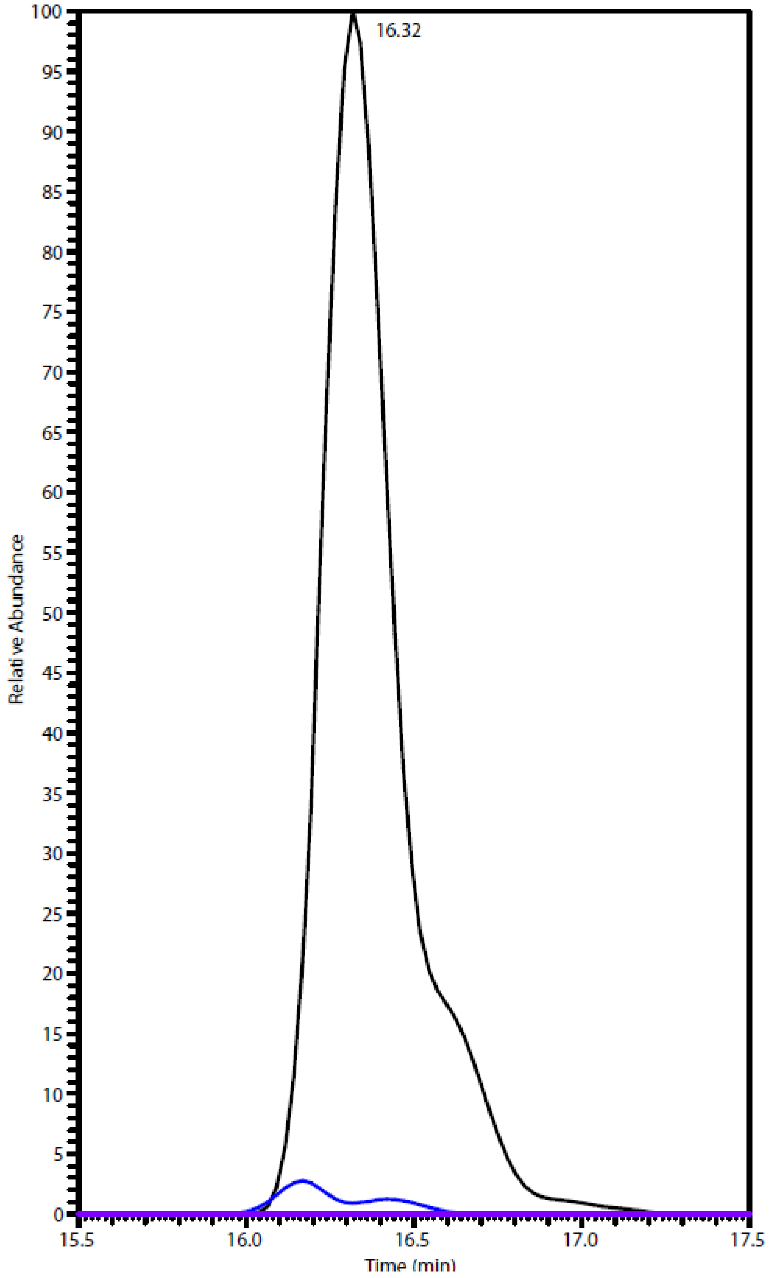

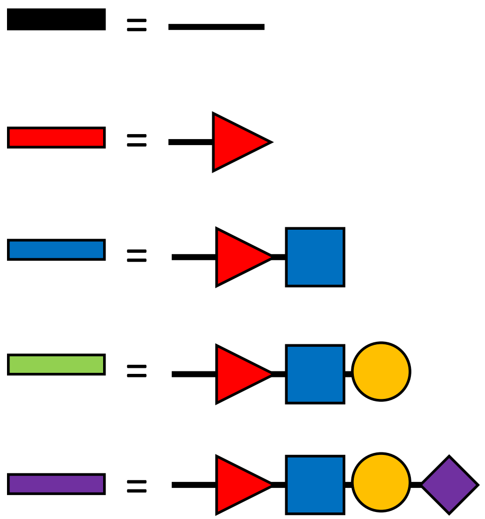

Figure S6E

EGF 12 : 274IDVNECVSNPCQDNATCLDQIGE296

#	a calc.	a obs.	b calc.	b obs.	b-18 calc.	b-18 obs.	b++ calc.	b++ obs.	Seq.	y calc.	y obs.	y++ calc.	y++ obs.	#
1	86.0964		114.0913		96.0808		57.5493		I					23
2	201.1234		229.1183		211.1077		115.0628		D	3766.441		1883.724		22
3	300.1918		328.1867		310.1761		164.597		V	3651.414		1826.21		21
4	414.2347		442.2296	442.2247	424.2191		221.6184		N	3552.345		1776.676		20
5	543.2773		571.2722	571.2734	553.2616		286.1397		E	3438.302		1719.655		19
6	703.3079		731.3028	731.302	713.2923		366.1551		C	3309.26		1655.134		18
7	802.3763	802.3683	830.3713	830.3674	812.3607		415.6893		V	3149.229		1575.118		17
8	1315.546		1343.541		1325.53		672.274		S	3050.161		1525.584		16
9	1429.589		1457.584		1439.573		729.2954		N	2536.991		1268.999		15
10	1526.641		1554.636		1536.626		777.8218		P	2422.948		1211.978		14
11	1686.672		1714.667		1696.656		857.8371		C	2325.896		1163.451		13
12	1814.731		1842.726		1824.715		921.8664		Q	2165.865		1083.436		12
13	1928.774		1956.769		1938.758		978.8879		N	2037.806		1019.407		11
14	2043.801		2071.795		2053.785		1036.401		D	1923.763		962.3853		10
15	2114.838		2142.833		2124.822		1071.92		A	1808.737		904.8719		9
16	3018.171		3046.166		3028.155		1523.587		T	1737.699		869.3533		8
17	3178.201		3206.196		3188.186		1603.602	1603.632	C	834.3662	834.3641	417.6867		7
18	3291.286		3319.28		3301.27		1660.144		L	674.3355	674.3344	337.6714		6
19	3406.312		3434.307		3416.297		1717.657		D	561.2515	561.2486	281.1294		5
20	3534.371		3562.366		3544.355		1781.687		Q	446.2245	446.2223	223.6159		4
21	3647.455		3675.45		3657.439		1838.229		I	318.166	318.1636	159.5866		3
22	3704.477		3732.472		3714.461		1866.739		G	205.0819		103.0446		2
23									E	148.0604		74.5339		1

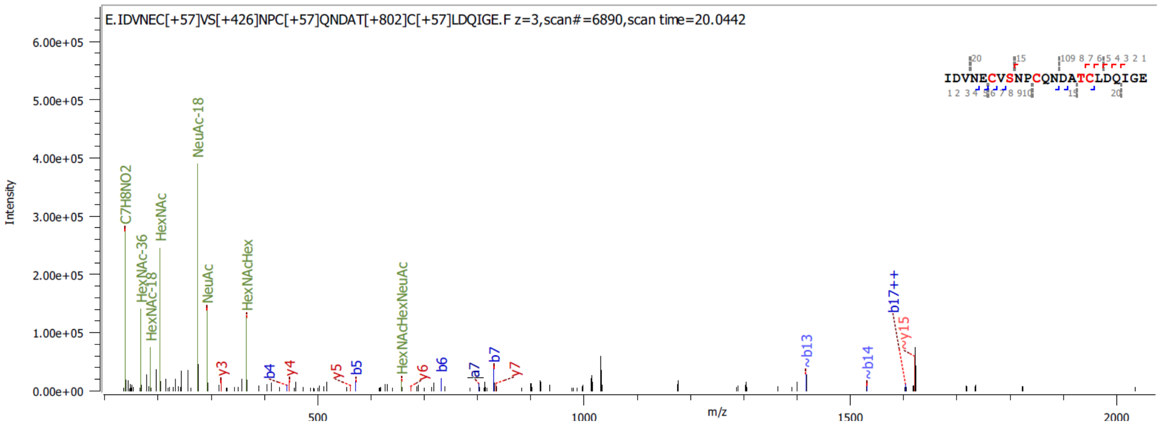

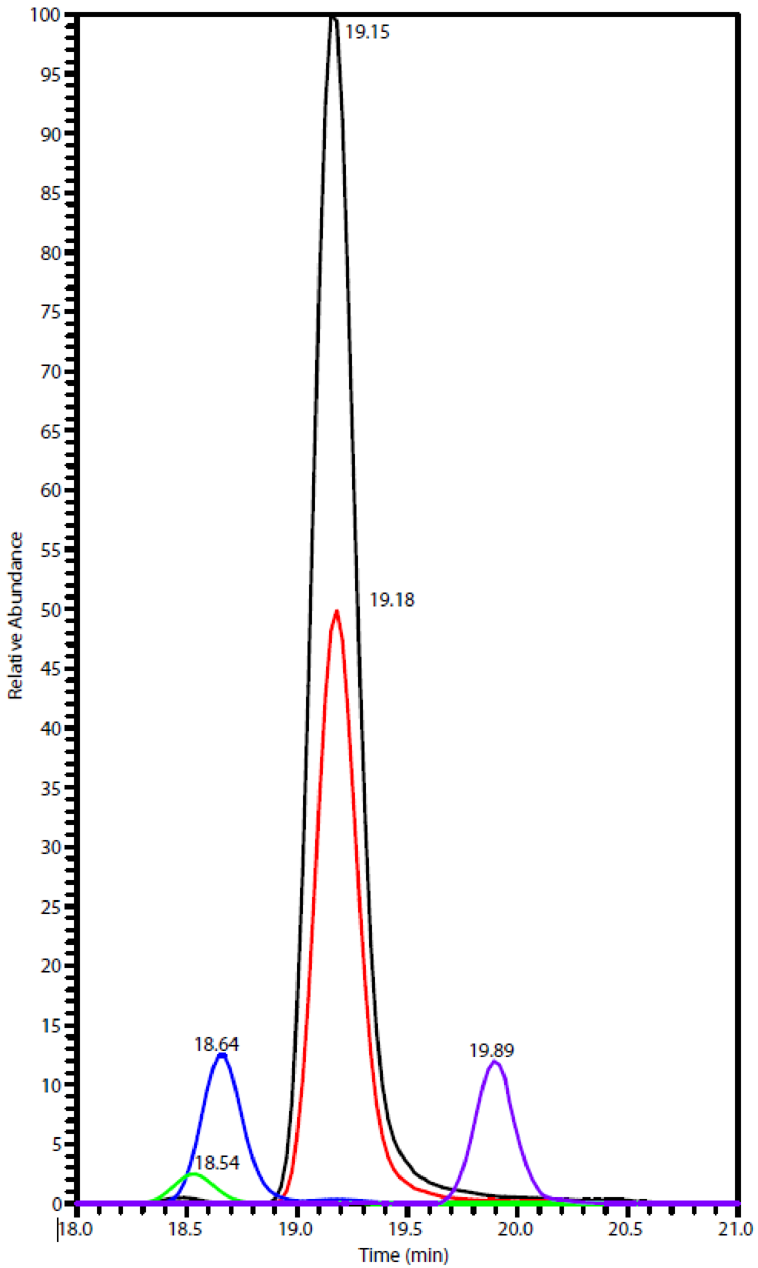

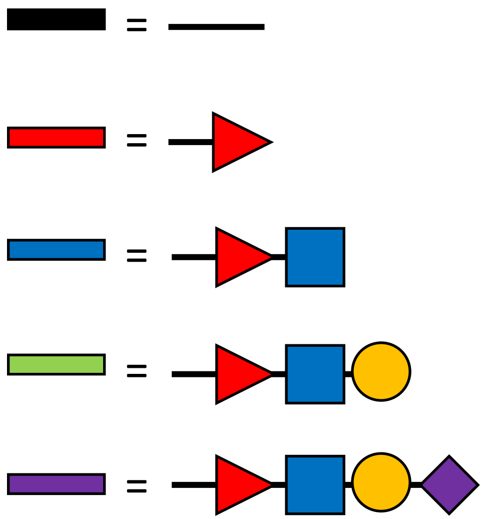

Figure S6. MS/MS spectra for *O*-fucosylated peptides from EGF6, 8, 9, 10 and 12. N1 EGF5-14 was co-expressed in CHO cells with LFNG, purified from media, reduced/alkylated, digested with proteases, and the resulting peptides were analyzed by nano-LC-MS/MS as described in Experimental Procedures. Peptides are shown from EGF6 (A), EGF8 (B), EGF9 (C), EGF10 (D), and EGF12 (E). The position of the *O*-glycans in a monosaccharide form was added to the sequence of each EGFs. The red triangle symbolizes the position of *O*-fucose, the blue circle the position of *O*-glucose, and the empty triangle indicates an *O*-fucosylation site added by the mutation but absent in the wild-type protein. The EIC shows relative amounts of the *O*-fucose glycoforms of the peptide. The key to the right indicates the color of the lines for each glycoforms. Asterisks denote ions that have a similar mass, but MS/MS fragmentation does not match the indicated peptide. Data can be found in Supporting Information Tables S3 to S22.
