# Supplementary material for: Cancer-associated Notch receptor variants lead to O-fucosylation defects that deregulate Notch signaling
Source: J Biol Chem. 2022 Oct 18;298(12):102616. doi: 10.1016/j.jbc.2022.102616 (PMC9672452; doi:10.1016/j.jbc.2022.102616)
Supplement: Table S24 [file mmc23.docx]

**List of synthesized DNA fragment for hNotch1 mutants construct**

The mutated site is shown in bold red and underlined

>G230R(246bp)

GCCACGGAGGCACCTGCCACAACGAGGTCGGCTCCTACCGCTGCGTCTGCCGCGCCACCCACACTGGCCCCAACTGCGAGCGGCCCTACGTGCCCTGCAGCCCCTCGCCCTGCCAGAAC**A**GGGGCACCTGCCGCCCCACGGGCGACGTCACCCACGAGTGTGCCTGCCTGCCAGGCTTCACCGGCCAGAACTGTGAGGAAAATATCGACGATTGTCCAGGAAACAACTGCAAGAACGGGGGTGCCT

>G309R (177bp)

TGTACCGAGGATGTGGACGAGTGCCAGCTGATGCCAAATGCCTGCCAGAAC**C**GCGGGACCTGCCACAACACCCACGGTGGCTACAACTGCGTGTGTGTCAACGGCTGGACTGGTGAGGACTGCAGCGAGAACATTGATGACTGTGCCAGCGCCGCCTGCTTCCACGGCGCCACCTGC

>G310R (177bp)

TGTACCGAGGATGTGGACGAGTGCCAGCTGATGCCAAATGCCTGCCAGAACGGC**A**GGACCTGCCACAACACCCACGGTGGCTACAACTGCGTGTGTGTCAACGGCTGGACTGGTGAGGACTGCAGCGAGAACATTGATGACTGTGCCAGCGCCGCCTGCTTCCACGGCGCCACCTGC

>T311P (177bp)

TGTACCGAGGATGTGGACGAGTGCCAGCTGATGCCAAATGCCTGCCAGAACGGCGGG**C**CCTGCCACAACACCCACGGTGGCTACAACTGCGTGTGTGTCAACGGCTGGACTGGTGAGGACTGCAGCGAGAACATTGATGACTGTGCCAGCGCCGCCTGCTTCCACGGCGCCACCTGC

>G347S(177bp)

TGTACCGAGGATGTGGACGAGTGCCAGCTGATGCCAAATGCCTGCCAGAACGGCGGGACCTGCCACAACACCCACGGTGGCTACAACTGCGTGTGTGTCAACGGCTGGACTGGTGAGGACTGCAGCGAGAACATTGATGACTGTGCCAGCGCCGCCTGCTTCCAC**A**GCGCCACCTGC

>T349P(177bp)

TGTACCGAGGATGTGGACGAGTGCCAGCTGATGCCAAATGCCTGCCAGAACGGCGGGACCTGCCACAACACCCACGGTGGCTACAACTGCGTGTGTGTCAACGGCTGGACTGGTGAGGACTGCAGCGAGAACATTGATGACTGTGCCAGCGCCGCCTGCTTCCACGGCGCC**C**CCTGC

>N386T (293bp)

TGTACCGAGGATGTGGACGAGTGCCAGCTGATGCCAAATGCCTGCCAGAACGGCGGGACCTGCCACAACACCCACGGTGGCTACAACTGCGTGTGTGTCAACGGCTGGACTGGTGAGGACTGCAGCGAGAACATTGATGACTGTGCCAGCGCCGCCTGCTTCCACGGCGCCACCTGCCATGACCGTGTGGCCTCCTTCTACTGCGAGTGTCCCCATGGCCGCACAGGTCTGCTGTGCCACCTCAACGACGCATGCATCAGCAACCCCTGTAACGAGGGCTCCA**C**CTGCGACAC

>D464N (308bp)

TCCTTCGAGTGCCAGTGTCTGCAGGGCTACACGGGCCCCCGATGCGAGATCGACGTCAACGAGTGCGTCTCGAACCCGTGCCAGAAC**A**ACGCCACCTGCCTGGACCAGATTGGGGAGTTCCAGTGCATCTGCATGCCCGGCTACGAGGGTGTGCACTGCGAGGTCAACACAGACGAGTGTGCCAGCAGCCCCTGCCTGCACAATGGCCGCTGCCTGGACAAGATCAATGAGTTCCAGTGCGAGTGCCCCACGGGCTTCACTGGGCATCTGTGCCAGTACGATGTGGACGAGTGTGCCAGCACCCCCTG

>A465T(308bp)

TCCTTCGAGTGCCAGTGTCTGCAGGGCTACACGGGCCCCCGATGCGAGATCGACGTCAACGAGTGCGTCTCGAACCCGTGCCAGAACGAC**A**CCACCTGCCTGGACCAGATTGGGGAGTTCCAGTGCATCTGCATGCCCGGCTACGAGGGTGTGCACTGCGAGGTCAACACAGACGAGTGTGCCAGCAGCCCCTGCCTGCACAATGGCCGCTGCCTGGACAAGATCAATGAGTTCCAGTGCGAGTGCCCCACGGGCTTCACTGGGCATCTGTGCCAGTACGATGTGGACGAGTGTGCCAGCACCCCCTG
